# Personalized medicine—concepts, technologies, and applications in inflammatory skin diseases

**DOI:** 10.1111/apm.12934

**Published:** 2019-05-24

**Authors:** Thomas Litman

**Affiliations:** ^1^ Department of Immunology and Microbiology University of Copenhagen Copenhagen Denmark; ^2^ Explorative Biology, Skin Research LEO Pharma A/S Ballerup Denmark

**Keywords:** Atopic dermatitis, endotypes, immunology, inflammatory skin diseases, personalized medicine, precision medicine, psoriasis, targeted therapy

## Abstract

The current state, tools, and applications of personalized medicine with special emphasis on inflammatory skin diseases like psoriasis and atopic dermatitis are discussed. Inflammatory pathways are outlined as well as potential targets for monoclonal antibodies and small‐molecule inhibitors.

## Introduction – why?

*One size does not fit all!* Or does it? For treatment of many – if not most – inflammatory skin conditions, the dermatologists’ first choice over the last 50+ years has been a topical glucocorticoid 1, most often yielding astonishing anti‐inflammatory effects: rapid relief of itch and ease of rash, bringing the inflamed skin back to a ‘near‐normal’ state within a few days (Box 1).

Box 1 **Size matters**

*One size fits all*, the paradigm of traditional medicine.

*One size does not fit all*, a mantra of personalized medicine, the goal of which is to provide ‘The right dose of the right drug for the right indication for the right patient at the right time.’ This is another mantra of personalized medicine, a much publicized quote ascribed to former FDA Genomics associate director, Felix Frueh, when he captured the essence of personalized medicine at the Annual FDA Science Forum in 2005 2. A variant of the above principles can be found in the ‘5R framework’ for improving research and development productivity in the pharma industry, with focus on ‘right target, right tissue, right safety, right patients, and right commercial potential’ 3.

But if such an efficient, universal, and inexpensive treatment is already available, where, then, is the unmet need for personalized medicine and targeted therapy? One may even argue that treatment with glucocorticoids *is* targeted therapy! Because glucocorticoids specifically bind to their molecular target, the cytosolic glucocorticoid receptor, and thereby induce downstream anti‐inflammatory effects. These effects are brought about via several mechanisms: non‐genomic direct activation of anti‐inflammatory proteins, DNA‐dependent (genomic) induction of anti‐inflammatory proteins, and protein interference (via transcription factors, such as NF‐κB) causing repression of inflammatory proteins 4, 5. Now, as glucocorticoid receptor activation produces pleiotropic (multiple and diverse) effects, and because the receptor is universally expressed – albeit to a varying degree – in most cell types, this accounts both for the high anti‐inflammatory efficacy, the broad mode of action, and for the adverse effects associated with – in particular: long‐term – glucocorticoid treatment. One such major adverse effect is skin *atrophy*, possibly mediated by the glucocorticoid receptor chaperone FKBP51 6, but also *systemic* side‐effects are observed, such as suppression of the hypothalamus‐pituitary‐adrenal (HPA) axis, due to percutaneous glucocorticoid absorption 7. Moreover, if large areas of the skin are covered with lesions, topical treatment is not a feasible solution. Therefore, and because of extensive disease heterogeneity – not all patients (especially, those with severe disease) respond to glucocorticoids, and all patients differ with respect to their genetic makeup – there is still a need for better, and more targeted therapy. In particular, the two most common inflammatory skin diseases, atopic dermatitis (AD) and psoriasis (PSO), have both a complex pathogenesis including several pathophysiological mechanisms 8, and a multitude of clinical manifestations 9, 10, which make them exemplary diseases for a personalized medicine strategy calling for improved stratification, development of targeted treatment, and prevention 11, 12.

Often, the term ‘personalized medicine’ is used synonymously and sometimes confused with precision/stratified/individualized/tailored/P4 medicine, targeted therapy, and pharmacogenomics. Here, I will mainly use ‘personalized medicine’, though, for clarity, the conceptual nuances of this and its related terms are summarized in Box 2.

Box 2 **WHAT? The different flavors of personalized medicine**

Numbers in parentheses correspond to count of Google hits as per February 19th 2019

Both American ‘‐ized’ and British ‘‐ised’ spellings have been included.

**Personalized medicine**

(5.2M) is an approach to both *preventive* care (e.g. identifying genetic risk factors to guide behavioral changes and preventive treatment, such as statins for hypercholesterolemia) and to drug *therapy* (e.g. early and accurate diagnostic tests that can guide targeted treatment and diminish side‐effects) based on the individual's genetic (and other relevant) information.

The term ‘personalized medicine’ – albeit with a slightly different, ethical connotation – can be found already in a 1971 article by W.M. Gibson, who envisages the family practitioner's role as a scientist‐physician who ‘Within a few years will likely have available to him a computer programmed for medicine providing him with a great store of knowledge literally at his fingertips’ 13. Thus, in the early years, personalized medicine focused on the ethical dimensions of patient‐centered practice 14. But actually, the foundation for personalized medicine can be traced all the way back to **Hippocrates** (460–370 BCE), who famously said ‘It's far more important to know what person the disease has than what disease the person has’, thus introducing the patient‐centric concept 15. Interestingly, today, such *patient‐centricity* is widely embraced by the pharma industry, which is increasingly engaging in a dialog with patients during the drug development process 16.

Due to concern that ‘personalized medicine’ can be misinterpreted as implying that a unique treatment can be designed for each individual, the National Research Council preferred the term ‘precision medicine’ in their 2011 report *Toward Precision Medicine* 17.

**Precision medicine**

(5.5M) is defined as ‘tailoring of medical treatment to the individual characteristics of each patient’. 17 But this does not mean that drugs are being developed uniquely for a patient, rather, it means that individual patients can be classified into subpopulations that differ in their response to a specific treatment. Thus, the focus is on identifying which treatments will work for which patients based on their individual genetic – and epigenetic – characteristics (for example, treatment of breast cancer patients with herceptin will only work for patients that overexpress HER2). An issue with the term precision, however, is that interpreted technically, it is a measure of statistical variability, and as such, it can be argued that medicine is not precise 18.

**Targeted therapy**

(3.6M) is often used synonymously with *molecularly targeted therapy, molecular medicine*, and *biologic therapy*, mainly to distinguish it from traditional chemotherapy in the context of cancer treatment. However, targeted therapy is neither limited to cancer, nor to biologics, as today, both small molecules and monoclonal antibodies are used in the targeted treatment of a wide variety of diseases, including asthma, atopic dermatitis, and psoriasis. The target concept is an old one and marks the beginning of modern pharmacology; it was developed by Paul Ehrlich around 1900, when he was studying antibodies and envisioned a hypothetical drug that would reach and kill its target (microbe) without harming the host; the *magic bullet* (German: Zauberkugel) 19. Indeed, today Ehrlich's vision has become a reality, where numerous highly specific monoclonal antibody‐based therapies are being applied or are in clinical development.

**Pharmacogenomics**

(2.9M) refers to the study of how genes affect an individual's response to drugs. The term is a combination of pharmacology and genomics, with the aim of developing safe and effective treatments. When it is applied to the study of drug metabolism, it is largely termed *pharmacogenetics*, while pharmacogenomics is a broader term encompassing all genes that may impact drug response 20. A typical use includes identification of fast and slow metabolizers due to single nucleotide polymorphisms (SNPs) in the CYP450 system, where the former will achieve suboptimal drug levels, while the latter will have increased risk of adverse drug reactions, and in worst case, death 21.

**Individualized medicine**

(357K) is the term preferred by Eric Topol (founder and director of the Scripps Translational Science Institute), mainly because it relates both to the medicine and the medical information – including both omics and digital technology – that is particularized to an individual, and because it is supposedly less ambiguous compared to the terms personalized and precision medicine 22. Note, however, that individualized medicine can also be understood as ‘truly’ individualized, such as a cancer vaccine based on the patient's particular tumor. In this respect, individualized medicine lies in one end of the therapeutic continuum, empirical medicine is at the other end, while the field of stratified medicine lies in between 23.

**Stratified medicine**

(112K) aims at matching a therapy with a specific patient population – who will have a therapeutically meaningful benefit of the treatment – by use of clinical biomarkers, which are, therefore, of utmost importance (e.g. as companion diagnostics, such as the FDA‐approved HercepTest that quantifies HER2, identifying patients who are likely to benefit from Herceptin), because they link the patient subpopulation with the therapy 23.

**P4 medicine**

(40K) stands for *predictive, preventive, personalized, and participatory* medicine. The term was coined by Leroy Hood (a pioneer of systems biology and co‐founder of the Institute for Systems Biology in Seattle) with special emphasis on the *participatory* part. The idea is that the digital revolution and rise of the Internet will empower consumers, who by their use of social media, mobile healthcare apps and wearables 24 generate the big data needed for systems medicine 25. Thus, Hood envisaged the emergence of a whole new healthcare system based on systems biology, big data, and networked consumers, who focus on both disease and wellness care, moving toward a holistic view on biological complexity.

**Tailored medicine**

(15K) emphasizes the move from the ‘one size fits all’ paradigm of traditional drug development and usage, to personalized medicine, where stratification of patient populations allows identification of responder subpopulations. One ethical issue with such an approach is that most participants in clinical trials in the US are white from higher socioeconomic levels, while ethnic minorities, who make up 40% of the population, are underrepresented. This disparity is problematic because certain diseases are more prevalent among ethnic minorities, who have a different genetic makeup and thus are likely to differ both in pathophysiology and response to treatment 26.

## From Disease Understanding to Biomarkers, Endotypes, and Targeted Treatment

### Theory

In theory, the logic is simple: If we can understand a disease, then we can also treat it. In particular, if we gain sufficient knowledge of its underlying molecular pathophysiology, then we can identify disease‐driving pathways and target relevant proteins. Or, better yet, it may be possible to take *preventive* measures even before the disease has manifested. Today, preventive medicine is made possible with the advent of new omics technologies, in particular, *next‐generation sequencing* (NGS) that enables determination of an individual's entire DNA sequence (six billion base‐pairs in a human diploid genome) in less than a day.^1^ For the 6000+ human (mostly rare) diseases caused by a single gene mutation, including the more than 600 known monogenic dermatoses 27, a correct molecular (genetic) diagnosis is crucial, both in terms of counseling and preventive measures [e.g. statin treatment of familial hypercholesterolemia) and in terms of avoiding ineffective and often stressful, even deadly treatments (such as cancer chemotherapy for multidrug resistant tumors 28]. In the best‐case scenario, it may even guide treatment. A striking example of such a case was recently reported for a seven‐year‐old boy suffering from a life‐threatening skin disease, junctional epidermolysis bullosa. After genetic analysis revealed the cause to be a splice‐site mutation in the LAMB3 gene, the patient was treated successfully with transgenic keratinocyte stem cells, which resulted in regeneration of the entire epidermis 29.

### Practice

In practice, most disorders are not as simple as that; they are polygenic, complex, and multifactorial, meaning that multiple genetic, epigenetic, lifestyle, and environmental factors play a role in the clinical manifestation of the disease. Such diseases include diabetes, cancer, and hypertension, as well as many inflammatory conditions, including asthma, inflammatory bowel disease (IBD), psoriasis, and atopic dermatitis. In these cases, a genomic ‘DNA fingerprint’ will give a *static* picture of the genetic susceptibility of an individual,^2^ but will not fully capture the *dynamic* nature of cells or diseases.

To this end, one needs to identify other relevant and robust *biomarkers* that reflect the various clinical phenotypes, and which eventually can form the basis for stratification of *endotypes*.^3^

Biomarkers for personalized medicine can be classified as *diagnostic*,* prognostic*, or *predictive*.

### Diagnostic biomarkers

Ideally, *diagnostic* biomarkers can detect diseases before they become symptomatic. Examples include early detection of prostate cancer by evaluation of serum prostate‐specific antigen (PSA) [albeit with relatively low sensitivity and specificity 35 and detection of other cancers by measuring circulating tumor cells in liquid biopsies 36]. But diagnostic biomarkers are more than just binary indicators of the absence or presence of disease. If they reflect the molecular pathology of the disease, then they may be able to precisely define and stratify its endotypes, and thus guide selection of the most effective targeted therapy. This also points to a need for improved *molecular disease taxonomy* 11, 37, because currently, for most diseases – including inflammatory skin diseases – endotypes are not incorporated in the WHO's latest revision of International Classification of Diseases, ICD‐11 38.

### Prognostic biomarkers

*Prognostic* biomarkers can, in principle, project the *disease trajectory*, i.e. indicate the likelihood of progression, remission, and future clinical events.^4^ In oncology, classical clinicopathologic biomarkers are tumor size, number of tumor‐positive lymph nodes, and distant metastases, which are used for staging and prognosis indication. In clinical trials, prognostic biomarkers are used to enrich for populations that are more likely to progress, as this increases statistical power and thus, reduces cost of drug development, and also guides decisions regarding the aggressiveness of the treatment 40.

### Predictive biomarkers

*Predictive* biomarkers are most important for guiding personalized medicine, because they have the potential to identify individuals that are more or less likely to respond to a given treatment. In clinical trials, predictive biomarkers are used to stratify the study population into biomarker positive (likely responders) and negative (non‐responders) patients, with the hope of meeting the clinical primary endpoint in the biomarker positive group 40.^5^ Examples of predictive biomarkers include polymorphisms in the cytochrome P450 superfamily [responsible for the hepatic – as well as extrahepatic – metabolism of most drugs, and thus, of immense importance for their pharmacokinetics 42] and variants of the human leukocyte antigen B (HLA‐B, associated with several hypersensitivity reactions, including toxic epidermal necrolysis); many more such gene variant–drug relationships can be found in *PharmGKB* (The Pharmacogenomics Knowledge Base, http://www.pharmgkb.org), a public, knowledge‐sharing resource that captures, curates, and integrates pharmacogenomics data, and which currently contains around 21 000 variant annotations and 132 PK/PD focused drug‐pathway diagrams 43. As targeted therapies, e.g. those based on monoclonal antibodies, are expensive and display variable response rates both in AD and PSO, it is important to identify and validate biomarkers for prediction of treatment outcome, as recently reviewed by Ovejero‐Benito et al. 44.

### Biomarker combinations

Because heterogeneous treatment responses can be due to a combination of factors, including disease complexity (multiple endotypes), genetic, epigenetic, and environmental effects, a *single* biomarker has only limited ability to capture all these aspects into a prediction of a patient's response to a given drug. Therefore, patient stratification may rely on the identification of *multiple* biomarkers, entailing multivariate statistical analysis and machine learning for finding the optimal linear and non‐linear biomarker *combinations* 45, i.e. those with highest sensitivity and specificity (maximizing the AUC of the ROC analysis) for a given classification. For example, we recently identified and validated a diagnostic miRNA classifier based on a linear combination of three miRNAs (miR‐155, miR‐203, and miR‐205) that could discriminate cutaneous T‐cell lymphoma (CTCL) from benign inflammatory skin diseases with 95% classification accuracy 46, 47. One advantage of using a biomarker panel as opposed to a single biomarker is that individual differences in the baseline levels of the biomarkers can be accounted for, in particular if the biomarkers of interest are normalized to a set of reference biomarkers. Other recent examples include a plasma protein (MMP‐2, sTNF‐R2, TSLP) panel for identification of ischemic stroke 48, a cell surface protein (CD25, CD64, CD69) panel for flow cytometric detection of sepsis 49, and a serum nuclear magnetic resonance (NMR) metabolomics‐derived biomarker (alanine, pyruvate, glycine, sarcosine) panel for early detection and grading of prostate cancer 50. For atopic dermatitis, Thijs et al. applied a panel of 147 serum biomarkers to stratify 193 AD patients into four main clusters, which may represent endotypes 51, although their analysis suggests that AD is very heterogeneous and may even reflect a disease spectrum rather than distinct endotypes.

Note, however, that the above examples only consider combinations of biomarkers of the *same* biological type or layer, such as genomic (DNA), transcriptomic (RNA), proteomic (proteins), or metabolomic (amino acids) markers. With the explosive development, both in omics technologies (summarized in Box 3) and in bioinformatics and computational tools, the natural next step is to move *out* of and *across (between)* the individual layers, *integrating* the various orthogonal (independent) biologic approaches in an integrative ‘multi‐omics’, systems biology strategy, also referred to as *integromics* 52, 53, 54. Conceptually, the layers in such an integrative approach can be compared to Google Maps (maps.google.com), which render a multilayered visualization of both *spatial* (locations, streets, landmarks) and real‐time *temporal* (traffic) data 22. And this is exactly the ambition of integromics^6^ on a human scale; to be able to visualize the various layers (mapping the genomic, epigenomic, transcriptomic, proteomic, metabolomic, etc. landscapes) of biologic pathways, as well as to be able to predict the dynamic effects of perturbations, such as targeting central molecules in the pathways, creating ‘roadblocks’ that will stop or diverge the traffic (information, signaling) flow in the system, to remain in the Google Maps analogy.

Box 3 **Omics technologies for integrative, personalized medicine**

**Genomics**

Next‐generation sequencing (NGS) is key to generating the vast amounts of DNA data for whole‐genome sequencing (WGS) and whole‐exome sequencing (WES) projects 55. Because the exome only comprises 1.5% (40 Mb) of our genome, it can be sequenced at a deeper coverage (>100× compared to 30×), faster (hours compared to days), and at lower price compared to WGS. However, it appears that most (80%) of the loci involved in complex diseases are located in the 98.5% noncoding – but important regulatory – regions of the genome 22. Therefore, and because the price of WGS continues to drop, it is today cost‐efficient to perform WGS for single‐nucleotide polymorphism (SNP) analysis, genotyping, pharmacogenomics and genome‐wide association studies (GWAS). If cost is a concern, then microarray‐ or bead‐based SNP analysis can be considered, albeit with considerably lower coverage than WGS.

A powerful tool to reveal the cellular complexity of in particular tumors 56, but also of individual genomic variation (mosaicism), is single‐cell sequencing, which is gaining momentum as the methodologies for whole‐genome amplification (WGA) and sequencing mature 57. This technique (as well as scRNA‐seq, see below) can also be used to profile T‐ and B‐cell receptor repertoires at the single‐cell level, thus enabling a full picture of the immune landscape and its dynamics 58.

**Transcriptomics**

Since the late 1990s, global gene expression analysis has been performed by use of microarrays 59. Today, due to improvements in next‐generation sequencing (NGS) technology (longer and more reads) and a concomitant drop in price,^7^ RNA sequencing (RNA‐seq) is the preferred method for transcriptomic profiling 60. A major advantage of quantifying gene expression is that it reflects the *dynamics* of the cellular system under investigation. This is also a major caveat, because what is measured is a *snapshot* of the transcriptome, which tends to vary extensively with time and space. Thus, when comparing transcriptomic profiles of biologic samples it is of utmost importance that the experimental conditions are as standardized as possible (a notion that also applies to proteomics, and, in particular, metabolomics); i.e. the specimens should be comparable, both with respect to *location* (more on this later, with special reference to skin biopsies), and timing, including sampling time and time from sampling to freezing and RNA extraction. Optimally, a *time course* experiment (multiple samples taken at different time points) should be performed to investigate the expression profiles’ temporal dependence 60, 61, which is also of importance for the selection of biomarkers, as some may display transient expression, while others are more stable, and therefore more robust in a clinical setting. Just as for DNA sequencing, single‐cell RNA‐sequencing (scRNA‐seq) is now opening a window to the cellular phenotype, as it allows for unprecedented detail analysis of cellular heterogeneity and development 62, 63. Finally, novel *in situ* sequencing techniques such as fluorescent *in situ* sequencing of RNA (FISSEQ) 64 and STARmap 65 allow for determination of the actual, 3‐dimensional location of gene expression in cells and tissues 66.

**Epigenomics**

At least three types of epigenetic systems co‐exist: DNA methylation, histone modification, and noncoding RNA (ncRNA, including miRNA, lncRNA, snoRNA, and many more).

DNA methylation is typically assessed by bisulfite treatment of the DNA – converting non‐methylated C's to U's, while methylated C's are protected from this conversion – followed by either microarray analysis or WGS (which captures all 29 million CpGs in the human genome, albeit at 10 times the cost of methylation arrays) enabling epigenome‐wide association studies (EWAS; 67). The interpretation of such studies, however, can be difficult, in particular if the starting material contains a mixture of different cell types, each with their own, highly cell‐type specific epigenome. Thus, it is necessary to perform cell‐type specific deconvolution of the signal in order to identify relevant epigenetic changes rather than just a shift in proportion of cell types 66, 68.^8^

For studying the ‘histone code’, genome‐wide histone modification assays apply chromatin immunoprecipitation (ChIP) and histone modification‐specific antibodies (to precipitate the DNA–histone complexes), followed by NGS (ChIP‐seq) to identify the bound DNA fragments. This has resulted in mapping of several human epigenomes 69 with promise for identification of epigenetic biomarkers 70 and with implications for epigenetic drugs, such as histone deacetylase (HDAC) inhibitors 70, 71.

Numerous microRNAs have already been identified – by microarrays, qRT‐PCR, and small RNA‐seq – as potential diagnostic, prognostic, and predictive biomarkers in cancer 72, 73, 74, 75, 76, 77, diabetes 78, and many other diseases, including inflammatory skin conditions like psoriasis 79, 80 and atopic dermatitis 81, 82. What remains to be seen is the potential of an emerging class of ncRNA, namely the long ncRNA (lncRNA, of which around 16 000 human variants have been found so far), in precision medicine, cancer 83 and inflammatory diseases 84.

**Proteomics**

Studying the proteome–by various mass spectroscopy methods – is important because gene expression levels are only approximations of the corresponding protein levels 85. Firstly, because not all mRNA is translated into protein – sometimes because miRNAs bind to the 3′UTR of their target genes, thus blocking translation 85, 86, and secondly, because post‐translational modifications, such as phosphorylation/dephosphorylation, are important determinants of protein function, which is why phospho‐proteomics is a relevant measure of protein function and dynamics of cellular signaling 87.

**Metabolomics**

The analysis – either by NMR or GC–MS – of the complete set of small‐molecule intermediates, including lipids (the lipidome, a subset of the metabolome) in a biological sample, provides a sensitive snapshot of its physiology, and can thus guide discovery of biomarkers 53, 87, 88. Application examples include ‘breathomics’, breath‐based metabolomics, where quantification of volatile organic compounds has diagnostic potential 89, urine metabolite‐based diagnosis of urinary tract symptoms 90, as well as assessment of glucocorticoid‐induced changes of the lipid profile of human skin 91. In particular, when combined with other – orthogonal – omics technologies, one can obtain mechanistic insight, e.g. on metabolic and inflammatory pathways 53.

**Glycomics**

The study of glycans (polysaccharides) includes analysis of glycosylated proteins (glycoproteins) and lipids (glycolipids), mainly by MS or HPLC. Since most human proteins are glycosylated, and glycans play important roles in many cellular processes, including cell adhesion, trafficking, and inflammation, individual variations in glycosylation patterns may serve as biomarkers for disease risk and response to therapy 92, 93. For example, heterogeneity in N‐glycosylation of immunoglobulin G (IgG) can modulate its inflammatory effect, with implications for regulation of the immune system 94.

**Phenomics**

A detailed description of the *phenome*, i.e. an account of the phenotypic traits of an organism, is crucial for building the translational bridge from genome‐scale biology to disease understanding, i.e. for establishing the genotype–phenotype relationship 95. In practice, it entails *deep phenotyping* of individuals, including collection of multidimensional clinical data (e.g. biochemical tests, pathology reports, physical examination, family history, demographics, and imaging), and importantly, a precise, comprehensive, and standardized description (metadata) of such data. This makes the data accessible and searchable and facilitates its integration with omics data for translation into disease endotypes and eventually, personalized medicine 96. To aid in connecting genomics and phenomics, a formal ontology (standardized vocabulary and annotation of phenotypes and relations to diseases) has been proposed by The Human Phenotype Ontology (HPO) project 97, which today links more than 13 000 phenotypic terms and over 156 000 disease annotations. Additionally, phenomics can be applied for construction of large‐scale *disease trajectories* based on information on *comorbidities* pulled from real‐world data (RWD) 98, such as observational data from disease registries and electronic health records (EHR). One such study used the Danish National Patient Registry (covering the whole population of Denmark, 6.2 million patients followed over 15 years) for generation of disease trajectories that can prove useful for predicting (and ultimately, preventing) disease progression of individual patients 99.

**Microbiomics**

A growing research field, initiated by the human microbiome project 100, and with potential for personalized medicine is the study (by NGS) of our *microbiome*, which is the sum of microorganisms (bacteria, archaea, fungi, and viruses) in and on our body (skin, mouth, nose, lung, gut, and vagina). In particular, the gut microbiome has been extensively researched and shown to play an important role in nutrition, metabolism, immune function, and numerous diseases, including inflammatory bowel disease (IBD), type II diabetes, cardiovascular disease, asthma, atopy 101, 102, and autism 103. The microbiome is also implied in drug interactions – studied by pharmacomicrobiomics 104, 105 – and e.g. digoxin has been shown to be metabolized and inactivated by specific gut bacteria 106. In the context of chronic, inflammatory skin diseases, dysbiosis of the skin microbiome has been associated with both PSO 107 and AD 108, 109. This opens possibilities for targeted, preventive intervention, such as administration of prebiotics (non‐digestible food components, like fibers) and probiotics (live microorganisms, such as Lactobacillus strains). Notably, the microbiome is *dynamic*; it undergoes temporal (e.g. circadian) and spatial fluctuations, both in composition and metabolic activity 110. The question of composition is addressed by targeted 16S rDNA taxonomic profiling and by – more comprehensive – *metagenomics* shotgun strategies (whole‐metagenome sequencing). But to capture the true dynamics of the microbiome, a full functional analysis must include both *metatranscriptomics* and *metabolomics*. The former addresses the question of which genes are expressed (collectively by the microbiome at a given time and condition), while the latter provides important information on which metabolites (both microbiota‐ and host‐derived) are present and interplay at the host–microbiome interface 111.

**Exposomics**

Genetic factors alone explain only a fraction of what we consider genetic diseases, including cancer 112. The remainder, perhaps more than 90%, can be attributed to environmental factors, also known as the exposome. The term *exposome* was coined by CP Wild in 2005, who broadly defines it as ‘every exposure to which an individual is subjected from conception to death’ 113. It encompasses three domains: internal, specific external, and general external. The internal exposome consists of endogenous factors, including circulating metabolites, hormones, lipids, oxidative stress, and our microbiome 114. The specific external factors include radiation, infections, contaminants, pollutants, diet, medicine, tobacco, and alcohol, while the general external factors encompass socioeconomic status, education, stress, environment (urban/rural), and climate, among others. Thus, due to the diversity of the exposome, and because it is in constant flux, the challenge is to decide what (which biomarkers of exposure are available, if any) and when to measure 113. One approach has been to apply metabolomics on consecutive saliva samples, assessing the ‘saliva exposome’, as it is easy to collect and measure, and can be used to monitor individual health trajectories 115. Biomarkers of exposure also enable *exposome‐wide association studies* (EWAS)^9^ ^116^, ^117^, which have promise in the near future. Why? Because the digital revolution has opened for disruptive technologies, such as continuous, cloud‐based tracking of big data [such as the Internet of Things, IoT, with a plethora of physical devices that connect, collect, and exchange data for IoT‐enabled health care 118], generated by wearable, environmental monitors and biosensors coupled to our smartphones – a realization of the ‘quantified self’ 119.

**Integromics**

Also known as integrated/integrative omics, combine two or more omics layers in order to identify relevant overlaps between these. For example, a five‐layer approach may include genomics, epigenomics (three sublayers: DNA methylation, histone code, miRNA), transcriptomics, proteomics, and metabolomics, which coupled to phenotype (phenomics) data appears as an ‘obvious’ integrative omics approach, and one that we are currently exploring. However, so far, most published studies are limited to three layers, namely genomics–transcriptomics–proteomics, which will capture post‐transcriptional regulatory mechanisms, whenever there is discrepancy between gene and protein expression 52, but which do not take advantage of the orthogonal information that e.g. metabolomics adds to the (almost) full picture 52, 53. A major concern about integromics analysis and sharing of such big medical data is the difficult question regarding privacy and security 120, which needs to be solved before a massive open online medical (MOOM) repository can become a reality 22.

Next, let us see how the above considerations apply to personalized medicine in inflammatory skin diseases, with special emphasis on atopic dermatitis and psoriasis.^10^

### Personalized medicine in inflammatory skin diseases

See Box 4.

Box 4 **Basic characteristics of PSO and AD**

**Table:** 

	PSO	AD
ICD‐10 CM codes	L40 Psoriasis; L40.0 Psoriasis vulgaris, plaque PSO (90%) L40.1 Generalized pustular PSO (GPP, rare) L40.4 Guttate PSO (2%); L40.8 Other	L20.9 Atopic dermatitis, unspecified L20.8 Other atopic dermatitis
Epidemiology & Comorbidity	Affects 1–8% of the adult population 121, amounting to at least 130 million people worldwide. Two peaks in age of onset: 20–30 years and 50–60 years. PSO is a systemic condition with several serious comorbidities, including psoriatic arthritis (20–30%), inflammatory bowel disease, metabolic syndrome, and cardiovascular diseases 122.	Affects 10–25% of all children and 2–10% of the adult population 123, corresponding to at least 320 million people worldwide,^11^ and with wide regional variation 125. 85–95% of all cases begin before the age of 5 years 126 Prevalence has more than doubled within the last 50 years 127, which suggests environmental effects,^12^ including lifestyle changes – such as ‘Westernization’ and the hygiene hypothesis 128. AD is associated with other atopic diseases, including asthma (50% risk), food allergy (30% risk), and allergic rhinitis/hay fever (up to 75% risk), which underlines its systemic nature 127.
Disease burden	Overall, measured by disability‐adjusted life years (DALYs, excluding mortality; i.e. years of healthy life lost due to disease/disability), skin diseases are the fourth leading cause of disability worldwide 129. Due to the chronic and pruritic nature of both PSO and AD, they negatively impact quality of life (QoL) of most patients (and their families) and impose a major socioeconomic burden 130.	
Etiology	Unknown, but high heritability and numerous susceptibility loci suggest complex, polygenic predisposition combined with environmental triggering factors, autoantigens, and systemic inflammation 131.	Unknown, but high heritability and several susceptibility loci suggest a complex genetic disease including epidermal barrier dysfunction, immune dysregulation, and environmental triggers 132.
Risk factors and triggers	Family history (genetics, HLA‐Cw6), psychogenic stress, skin injury (Koebner phenomenon), streptococcal infections, medications, smoking, obesity 131.	Family history, FLG mutations, cold dry climate, irritants (detergents, wool), infections (S. aureus), allergens (house dust mites, pollen), cats 133, food allergens 132.
Pathogenesis	IL‐23/Th17 axis key driver 134 For details, see Fig. 1.	Th2 axis (IL‐4/IL‐13/IL‐5/IL‐31) dominating 134 For details, see Fig. 2.
Genetics	Concordance rate, monozygotic twins: 33% Concordance rate, dizygotic twins: 17% Heritability: 60–75% 135	Concordance rate, monozygotic twins: 44–86% Concordance rate among dizygotic twins: 10–23% Heritability: 69–86% 136
GWAS	HLA‐Cw6: strongest known risk allele, OR 4.32 126 Nine PSO susceptibility regions, PSORS1‐9, containing mostly immune‐related genes +60 PSO susceptibility regions 137	FLG: strongest known risk factor 138, more than 40 LOF mutations described 139 OR 1.61–1.92 140 31 susceptibility loci, most related to innate immune system 141 OR 0.90–1.14 (except for FLG) +70 gene variants (population‐specific) described 139, 142
Transcriptomics	+2600 DEG between lesional PSO and healthy skin 143 ~1800 DEG between lesional and non‐lesional skin 144	+1300 DEG between lesional AD and healthy skin 145 ~ 600 DEG between lesional and non‐lesional skin 146
Potential biomarkers	IL‐19 blood levels correlate with disease activity 147 IL‐2, IL‐5, IL‐10, IL‐12, IL‐22, GM‐CSF serum levels correlate with treatment effect 148 Skin transcriptome response to etanercept 149, ixekizumab 150, brodalumab 151, guselkumab 152, risankizumab vs ustekinumab 153	FLG stratifies for early‐onset persistent AD 154 IgE blood levels stratify for intrinsic/extrinsic AD 155 TARC (CCL17) in serum correlates with disease activity 155 IL‐31 levels associated with itch 155 IL‐33 serum levels correlate with disease severity 156 Skin transcriptome response to UVB 157, cyclosporin A 158, dupilumab 159, apremilast 160, fezakinumab (IL‐22) 161.
Top‐20 targets^13^	CARD14 TYK2 IL12B TRAF3IP2 JAK2 PDE4A ITGB2 TNF IL17RA IL17A VDR ERAP1 IL23R TNFAIP3 NOD2 JAK1 JAK3 IL23A CD2 NR3C1	IL13 FLG IL4R RXRA SPINK5 PPIA JAK2 FKBP1A CD2 NR3C1 VDR HRH1 CYSLTR1 JAK1 PLA2G7 IGHE RXRB PDE4B RARG RXRG
Current treatment guidelines	*Topical coal tar:* antipruritic, combined with UVB 162 *Topical corticosteroids*: anti‐inflammatory *Topical vitamin D analogues:* calcipotriol (often in combination with betamethasone dipropionate) inhibits epidermal hyperproliferation, induces differentiation, anti‐inflammatory *Topical salicylic acid:* keratolytic effect *Oral*: methotrexate, cyclosporin A, acitretin (for severe PSO), apremilast (PDE4 inhibitor), fumaric acid esters *Biologics:* etanercept, infliximab, adalimumab (TNF‐α); ustekinumab (IL‐12/IL‐23); secukinumab, ixekizumab (IL‐17A); brodalumab (IL‐17RA); guselkumab, tildrakizumab (IL‐23)	*Emollients:* for moisturizing the skin (lipid‐rich) *Antiseptics*: bleach (sodium hypochlorite 0.0005%) bath 163 *Topical corticosteroids*: anti‐inflammatory, relieve itch, e.g. hydrocortisone, betamethasone valerate, clobetasol *Topical calcineurin inhibitors*: tacrolimus (Protopic) or pimecrolimus (Elidel) *Oral calcineurin inhibitor:* cyclosporin A (severe AD) *Antibiotic creams*: to fight skin infections, e.g. fucidin/fucicort *Biologics (monoclonal antibodies), injectable*: targeted therapy, e.g. dupilumab (anti‐IL‐4R)

### Psoriasis

Psoriasis typically presents as thick, erythematous, scaly plaques due to hyperproliferation of keratinocytes. Therefore, it was originally considered an epidermal, keratinocyte‐specific disorder, and it was not until the mid‐1980s, when first, immunosuppression by cyclosporine 164 and later, bone marrow transplantation 165 resulted in remarkable clearance of psoriatic plaques that a major paradigm shift occurred, and psoriasis appeared as a Th1 cell driven, systemic disease 131. Another paradigm shift was precipitated by the discovery of a new T‐cell subset of IL‐23‐regulated IL‐17‐producing Th17 cells in the experimental autoimmune encephalomyelitis (EAE) mouse model 166. This, together with the findings of increased levels of Th17 cells 167 and of IL‐23, the ‘master’ regulator of Th17 development, in psoriatic lesions 168, identified psoriasis as a mixed Th1/Th17 disease.

**Figure 1:**
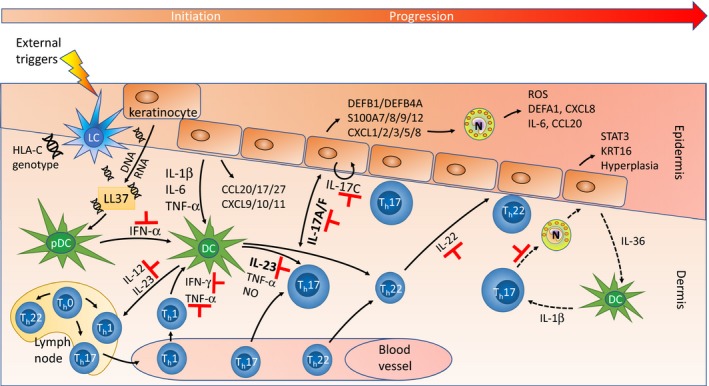
Pathways in the pathogenesis of PSO. Environmental triggers (e.g. drugs, infections, physical and psychological trauma) cause predisposed individuals to develop an autoimmune reaction, although the exact initiation mechanism is still poorly understood. One explanatory model 258 suggests that the autoantigen is LL37 (cathelicidin antimicrobial peptide, encoded by CAMP), which complexes with DNA and RNA released from stressed keratinocytes. This induces plasmacytoid dendritic cells (pDCs) to produce IFN‐α, which activates dermal dendritic cells (DCs). These cells migrate to skin‐draining lymph nodes, where they secrete IL‐12 and IL‐23, hereby stimulating naïve T‐cells to differentiate into T_h_1, T_h_17, and T_h_22 cells. The T_h_ cells are attracted into the dermis by chemokines (CCL20, CCL17, CCL27, CXCL9/10/11) released by keratinocytes. T_h_1 cells produce IFN‐γ and TNF‐α, while T_h_17 cells release IL‐22 and IL‐17 family cytokines. The latter (IL‐17A/F) trigger epidermal keratinocytes to a feed‐forward inflammatory response 169, inducing numerous psoriasis‐associated genes [defensins, S100 proteins, chemokines; keratinocytes also produce IL‐17 cytokines, shown is a putative, autocrine IL‐17C loop 175] and stimulating keratinocyte proliferation. The released chemokines CXCL1/2/3/5/8 recruit neutrophils (N), which generate ROS (reactive oxygen species), α‐defensin (DEFA1), CXCL8, CCL20, and IL‐6. IL‐23 (released by activated DCs) stimulates differentiation and expansion of T_h_22 cells, which secrete IL‐22 that induces STAT3 and KRT16 expression. This causes further epidermal hyperplasia and eventually formation of the psoriatic plaque. To the right (punctuated arrows) is shown the IL‐36/IL‐1 pathway prevalent in *pustular psoriasis*, which is characterized by accumulation of neutrophils; here, IL‐17 activated neutrophils trigger increased IL‐36 activity, which stimulates DC's to produce IL‐1β reinforcing the T_h_17 axis 179. Indicated with ⊣ are targets of approved and emerging drugs, most of which are monoclonal antibodies (see Table 2). Figure modified, mainly from van de Kerkhof & Nestle in 131, but also from Noda et al. 134, and Conrad & Gilliet 179.

Today, the central role of the IL‐23/Th17 inflammatory pathway in the immunopathogenesis of PSO (summarized in Fig. 1) is firmly established and has paved the way for development of novel targeted therapies that disrupt IL‐23/IL‐17 signaling (Fig. 1A) 12, 169. And the results are impressive: For moderate‐to‐severe plaque psoriasis, PASI 75^14^ was obtained for 75–91% of patients treated for 12 weeks with the IL‐17A antagonists ixekizumab or secukinumab, PASI 90 was reached for 54–73% 172, 173, while 78% and 53% of patients treated with the IL‐17RA inhibitor brodalumab achieved PASI 90 and PASI 100 (complete clearance), respectively, after 52 weeks 174. Brodalumab blocks signaling by the five IL‐17 dimers (IL‐17A/F/C/E/AF) through the IL‐17RA subunit (Fig. 3A). This causes inhibition of the downstream pleiotropic effects of IL‐17RA and probably explains the potentially higher clinical efficacy obtainable by receptor blockade compared to neutralization of a single ligand 175, 176, 177. In line with the efficacy of blocking downstream cytokine signaling are the impressive Phase II data on the TYK2 inhibitor BMS‐986165, where PASI75 was obtained for 75% of patients at week 12 178.

Different forms of PSO are associated with different pathways; *chronic plaque psoriasis* (also known as psoriasis vulgaris, the most common form) is dominated by the above‐mentioned IL‐23/Th17 pathway, while *acute, erythrodermic psoriasis* is characterized by Type I interferon (IFN‐α) producing plasmacytoid dendritic cells, and *pustular psoriasis* is associated with the IL‐36/IL‐1 pathway and accumulation of neutrophils^15^ ^179^. This heterogeneity in immunopathogenesis highlights the complexity of psoriasis, as well as provides guidance – by identification of biomarkers reflecting the different endotypes – for novel and optimized targeted therapies. These therapies include promising new modalities, such as bispecific (e.g. blocking both TNF‐α and IL‐17^16^) 180, 181, and even *trispecific* antibodies 182, as well as vaccines 183. A compilation of these new and emerging treatment options for psoriasis can be found in Table 2.

The basic characteristics of psoriasis are summarized in Box 4, of which the following are of particular relevance for personalized medicine:

#### Comorbidities

Because PSO is a systemic disease associated with multiple severe comorbidities, including psoriatic arthritis (PsA) and cardiovascular disease (CVD) 122, targeted treatment of e.g. the IL‐23/Th17 pathway may not only reverse the cutaneous manifestations of the disease, but also the systemic, inflammatory comorbidities.

#### Genomics

GWAS (genome‐wide association studies) have already identified more than 60 risk loci, including several psoriasis susceptibility regions (PSORS), most of which contain immune system related genes 137. The increasing amount of genomic data may allow for identification of new variants (endotypes) of PSO, possibly predict who will develop the disease, identify responders to specific drugs, and guide further development of targeted therapies.

#### Epigenomics

EWAS (epigenome‐wide association studies) on PSO are emerging 184 and a recent study on 39 Indian PSO patients suggested that differential DNA methylation (comparing lesional to non‐lesional skin) can regulate the expression of key genes involved in the pathogenesis of PSO 185. In addition to DNA methylation, *histone modification*, specifically methylation of H3K27 and H3K4, showed some promise as pharmacoepigenetic biomarkers in a study of psoriasis patients’ response to biologics 186. Common for both of the above cases is that larger independent validation cohorts are needed to confirm the initial findings. Finally, several inflammation‐associated miRNAs, such as miR‐146a, miR‐21, miR‐31, miR‐221, and miR‐222, are consistently found to be upregulated in PSO skin 187, 188 and may be useful as disease activity biomarkers.

#### Transcriptomics

Analysis of the mRNA profiles of lesional, non‐lesional, and healthy skin has identified more than 2000 differentially expressed genes (DEGs), many of which may serve as potential biomarkers for disease progression and response to therapy 189. For a compilation of such studies with links to the actual data, please see Table 1.

**Table 1:** Selected omics studies on psoriasis (PSO) and atopic dermatitis (AD)

GEO ID	Year	Dx	Focus (# samples)	Technology	Reference
GSE16161	2009	AD, PSO, NN	AD‐LS (9), PSO‐LS (15), NN (9)	HG‐U133_Plus_2	277
GSE32924	2011	AD, NN	LS (13), NL (12), NN (8)	HG‐U133_Plus_2	145
GSE27887	2011	AD	UVB, LS, NL, w0/w12, 10 pts. (35)	HG‐U133_Plus_2	157
GSE36842	2012	AD, NN	Acute/chronic, LS, NL, NN, 10 pts (39)	HG‐U133_Plus_2	196
GSE75890	2016	AD, PSO, NN	Mild ex‐/intrinsic, AD (14), PSO (9), NN (8)	HG 2.1 ST	278
GSE60709	2014	AD, NN	Epidermal shave, LS (12), NL (7), NN (14) DNA methylation, skin and blood	Illumina HT‐12V3.0 Infinium 27K	223
GSE107361	2018	AD	Infants/adults, LS (39), NL (40), NN (29)	HG‐U133_Plus_2	212
GSE58558	2014	AD	Cyclosporin A, LS, NL, w0/w2/w12 (109)	HG‐U133_Plus_2	158
GSE59294	2014	AD	Dupilumab, LS, NL, w0/w4 (40)	HG‐U133_Plus_2	159
GSE120721	2015	AD, NN	LCM, LS (15), NL (15), NN (22), epi/dermis	HG‐U133_Plus_2	222
GSE65832	2015	AD	RNA‐seq, LS (20), NL (20)	Illumina GA IIx	279
GSE81119	2017	‘AD’ mice	Mouse models of inflammation and ‘AD’ (37)	MG 1.0 ST	280
NA	2018	AD	Tape strip RNA‐seq, LS (11), NL (18), NN (13)	Ion Torrent	213
GSE120899	2018	AD	Apremilast, LS, NL, w0/w12 (59)	HG‐U133_Plus_2	Not published?
GSE99802	2018	AD	Fezakinumab, LS, NL, w0/w4/w12 (302)	HG‐U133_Plus_2	161
GSE121212	2019	AD, PSO, NN	RNA‐seq, AD (27LS, 27NL), PSO (28LS, 27NL), NN (38)	Illumina GA	334
GSE14905	2008	PSO, NN	LS (33), NL (28), NN (21)	HG‐U133_Plus_2	143
GSE13355	2009	PSO, NN	LS (58), NL (58), NN (64)	HG‐U133_Plus_2	281
GSE31037	2011	PSO, NN	miRNA, LS (24), NL (23), NN (20)	Illumina GA IIx	282
GSE30999	2012	PSO, NN	LS (85), NL (85)	HG‐U133_Plus_2	283
GSE26866	2012	PSO	LCM, LS (20), NL (17), epi/dermis,	HG‐U133_A 2.0	284
GSE11903	2009	PSO	Etanercept, LS, NL, w0/1/2/4/12 (89)	HG‐U133_Plus_2	149
GSE31652	2012	PSO	Ixekizumab, LS, w0/w4 (30)	HG‐U133_Plus_2	150
GSE55201	2014	PSO, NN	Ixekizumab, blood, LS, NN, w0/w2 (81)	HG‐U133_Plus_2	285
GSE51440	2014	PSO	Guselkumab, LS, NL, w0/w1/w12 (59)	HG‐U133_Plus_PM	152
GSE53552	2014	PSO	Brodalumab, LS, w0/w1/w2/wq6 (99)	HG‐U133_Plus_2	151
GSE69967	2016	PSO	Tofacitinib, LS, NL, d0/1/3/w1/2/4/12 (95)	HG‐U133_Plus_2	286, 287
GSE54456	2014	PSO, NN	RNA‐seq, LS (92), NN (82)	Illumina GA	288
GSE57225	2014	PSO‐AD/ACD	PSO (23), AD (10), ECZ (13), NL (16)	SurePrint G3 8x60K	289
GSE63741	2016	PSO, AD, other	AD‐LS, PSO‐LS, ACD, LP, NN (30 each)	PIQOR 2.0	290
GSE80047	2016	PSO, PPP(P)	PPP (3), PPPP (6), PSO (10), NN (31)	HG‐U133_Plus_PM	291
GSE79704	2017	PSO, GPP, NN	GPP‐LS (32), PSO‐LS (12), NN (20)	HG 2.1 ST	292
GSE73894	2017	PSO, NN	DNA methylation, LS (135), NL (41), NN (62)	Infinium 450k	293
GSE115797	2018	PSO	DNA methylation, LS (24), NL (24)	Infinium 450k	185

ACD, allergic contact eczema; Dx, diagnosis; d, day; epi, epidermis; ECZ, eczema (non‐atopic); GPP, generalized pustular psoriasis; LCM, laser‐capture microdissection; LP, lichen planus; PPP, palmoplantar pustulosis; PPPP (palmoplantar pustular psoriasis); PSO‐AD, patients co‐affected by both PSO and AD; w, week.

#### Microbiome

The cutaneous microbiome has been suggested as a factor that could trigger the immune system and initiate development of psoriasis 107, but as to date, the few and mainly descriptive data have been inconclusive. A recent analysis of the gut microbiome of 52 PSO patients suggested a specific ‘psoriatic core intestinal microbiome’ that differed from what is found in healthy subjects 190, but since the latter (healthy) data were pulled from the Human Microbiome Project, the analysis is confounded (with study), and calls for confirmation by a direct comparison of PSO patients with age and gender‐matched healthy controls. To establish – or rebut – a possible causative link between the microbiome (and its modulation by antibiotics, pre‐ or probiotics), psoriasis pathogenesis, and therapeutic effect, prospective, longitudinal intervention studies are needed. Also note that current microbiome analyses focus on taxonomic characterization (composition of the microbial community) rather than on functional, integrative studies involving *metatranscriptomics* and *metabolomics*, which eventually may enable in‐depth understanding of the dynamics of the microbiome 111.

### Atopic dermatitis

Atopic dermatitis is the most common chronic, relapsing inflammatory skin disorder, characterized by intense itch (pruritus), redness (erythema), and eventually, thickening (lichenification) of the skin due to chronic rubbing. It affects 10–25% of all children, most with onset before 2 years of age, and 2–10% of adults 123, with wide regional variation 125, and with a prevalence that has more than doubled over the last 50 years 127. Due to its chronic and pruritic nature, AD adversely affects the quality of life (QoL) of most patients, in particular due to sleep disturbance and skin infections, and is also often followed by other atopic diseases, such as food allergy, asthma and allergic rhinitis, known as the ‘atopic march’ 132. Note, however, that <10% of AD patients travel the full atopic march (i.e. clinical manifestation of all four comorbidities) and that the risk is highest in the early‐onset persistent AD phenotype 191, 192.

**Figure 2:**
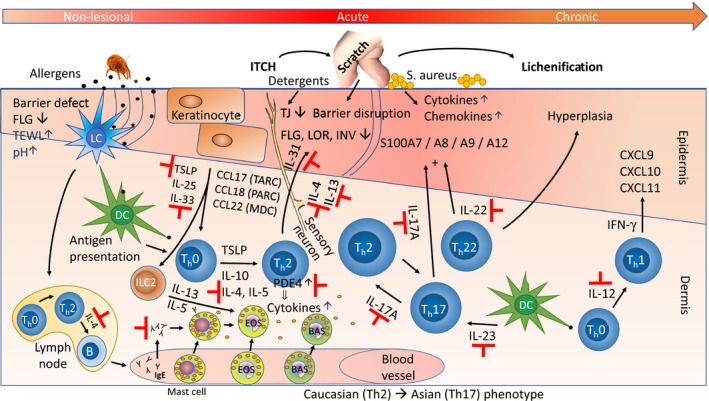
Pathways in the pathogenesis of AD. Epidermal barrier defects, which are partly due to FLG mutations, are associated with increased trans‐epidermal water loss [TEWL), increased skin pH, and penetration of epicutaneous allergens, such as dust mite debris. When the allergens encounter antigen‐presenting epidermal Langerhans cells (LCs, for an excellent review of the interplay between LCs and the epidermis, see Clayton et al. 259] and dermal dendritic cells (DCs), this causes immune activation and recruitment of inflammatory cells, including ILC2 [type 2 innate lymphoid cells 260] and type 2 helper T‐cells (T_h_2) that produce and release IL‐4, IL‐5, IL‐13, and IL‐31. These cells are considered part of the skin‐associated lymphoid tissue (SALT), the immunologically active cutaneous microenvironment, a concept which was proposed already in 1983 by Streilein 261. IL‐4 and IL‐13 suppress expression of terminal differentiation genes (such as FLG, LOR, INV), and also of tight junction (TJ) genes 208 leading to barrier disruption, while IL‐31 also acts directly on sensory neurons, triggering the itch–scratch cycle. This further damages the epidermis, increasing the risk of penetration by pathogens like Staphylococcus aureus. The stressed keratinocytes release TSLP, IL‐25, and IL‐33 that also drive T_h_2 differentiation. The T_h_2 cytokines induce IgE production in B cells and subsequently, release of inflammatory mediators (e.g. histamine) from activated (IgE bound) mast cells, basophils, and eosinophils. T_h_22 cells release IL‐22, which causes epidermal hyperplasia, and also, in synergy with IL‐17 – released from T_h_17 cells – induces expression of a subset of S100 family proteins. Acute AD lesions are characterized by a T_h_2 skewed (T_h_2, T_h_17, T_h_22) response, while chronic AD, which is often lichenified (thickened) by chronic scratching, progressively activates the T_h_1 axis with IL‐12 release, IFN‐γ expression and induction of chemokines (like CXCL9/CXCL10/CXCL11). Indicated with ⊣ are targets of approved and emerging drugs (see Table 2 for a detailed list). Figure modified, mainly from Vakharia & Silverberg 262, based on the original by Leung 2000 263 and 2004 264. For other representations, see Noda et al. 134, Paller et al. 265, Weidinger et al. 9, Lee et al. 9, 266, and Brunner et al. 267, 268.

The pathogenesis of AD is complex (illustrated in Fig. 2) and multifactorial as it involves genetic, immunologic, and environmental factors 193, including a defective skin barrier, permissive for entry of allergens that trigger inflammation, immune dysregulation with increased numbers of T‐cells and dendritic cells (DCs) and high levels of inflammatory molecules, and alterations in the cutaneous microbiome with overgrowth of Staphylococcus aureus. AD was first identified as a Th2 (IL‐4, IL‐13, IL‐31) driven disease 194, and later found to have also a Th22 (IL‐22) component 195 as well as variable Th17 and Th1 immune activation, the latter more pronounced in chronic AD 196.

**Figure 3:**
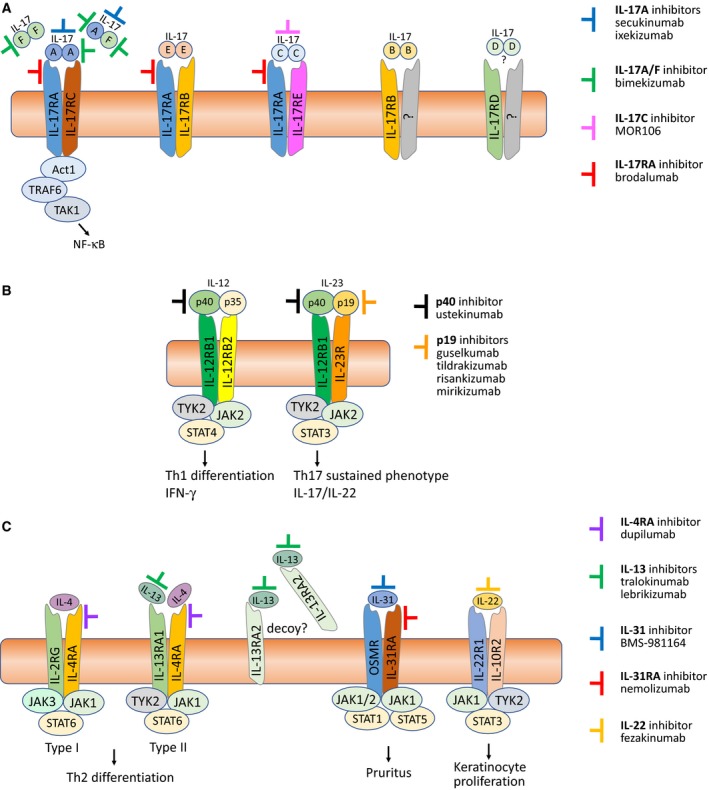
(A) Targeting the IL‐17 family of cytokines and their receptors. The six members of the IL‐17 cytokine family (IL‐17A/B/C/D/E/F) are shown as functional, disulfide‐linked homodimers, as well as the IL‐17A/F heterodimer 175. Also shown are their respective, heterodimeric receptors, each consisting of different combinations of five homologous receptor subunits (IL‐17RA/RB/RC/RD/RE). IL‐17A, IL‐17F (homodimers) and IL‐17A/F (heterodimer) signal through the IL‐17RA/RC receptor complex, IL‐17E (also known as IL‐25) via IL‐17RA/RB, IL‐17C via IL‐17RA/RE, while IL‐17B and IL‐17D signal via yet to be determined receptors. Indicated are also monoclonal antibodies that target either the cytokines or the IL‐17RA receptor subunit. Because IL‐17RA is common to signaling via IL‐17A/F/C/E/AF, blocking it will inhibit the downstream activities of all five IL‐17 dimers. IL‐17A/F and IL‐17RA inhibitors have already shown substantial effect in PSO, and currently, the IL‐17C inhibitor MOR106 is being tested in a Phase II clinical trial in moderate to severe AD 269. (B) Targeting IL‐12 and IL‐23. IL‐12 (p40/p35) and IL‐23 (p40/p19) are heterodimers that share the same p40 subunit. IL‐12 binds to the IL‐12Rβ1/β2 heterodimeric receptor and stimulates JAK2‐TYK2 to phosphorylate mainly STAT4, inducing IFN‐γ and a Th1 immune response. IL‐23 binds to the IL‐12Rβ1/IL‐23R heterodimeric receptor, and also induces JAK2‐TYK2 to phosphorylation, but primarily of STAT3, leading to Th17 signaling and release of IL‐17A/F and IL‐22 270. Because the p40 subunit is common to both IL‐12 and IL‐23, targeting it will inhibit the effects of both cytokines 271, while the p19‐specific antagonists target only the ‘master’ regulator of Th17 development, IL‐23 12. (C) Targeting IL‐4/IL‐13, IL‐31, and IL‐22. The two homologous cytokines, IL‐4 and IL‐13, drive type 2 inflammation and share many biological activities 272, the main differences being in their receptor interaction: The IL‐4R Type I receptor consists of the IL‐4RA and common‐gamma chain (IL‐2RG) subunits, and has IL‐4 as its exclusive ligand, while the IL‐4R Type II receptor is composed of the IL‐4RA and IL‐13RA1 chains, and binds both IL‐4 and IL‐13. The single‐chain IL‐13RA2 receptor is thought to function as a decoy receptor as it seems to lack the ability to induce intracellular signaling 273. As illustrated, targeting the common IL‐4RA subunit will inhibit the effects of both IL‐4 and IL‐13 signaling. IL‐31 signals via a heterodimer consisting of IL‐31RA and the oncostatin M receptor (OSMR), which is also common to oncostatin M (OSM), a member of the homologous IL‐6 superfamily 274. The IL‐31 receptor is found on sensory neurons in the dorsal root ganglia, where the itch sensation originates, which is why targeting IL‐31 by e.g. nemolizumab can potentially disrupt the itch–scratch cycle of pruritic diseases like AD 275. IL‐22 signals through the heterodimeric IL‐22R1/IL‐10R2 receptor and induces epidermal hyperplasia in AD, which is why the IL‐22 antagonist fezakinumab shows some promise in treatment of severe AD 161.

Compared to PSO, both our molecular disease understanding and treatment options for AD are lagging some 10–15 years behind 197. For example, the first FDA‐approved biologic for treatment of moderate‐to‐severe AD, namely the much touted IL‐4Ra inhibitor dupilumab, reports response rates in the range of 44–52% EASI 75^17^ ^200^, comparable to the rather moderate PASI75 response rates of the first generation TNF‐α targeting antibodies. Thus, there is still room for improvement and for development of even more efficacious targeted therapies; therapies that are tailored to the remaining subset(s) of severe AD patients, who may benefit from a personalized, endotype‐specific, treatment.

One reason that AD is a less‐mature field is the high diversity of the atopic landscape, with a wide spectrum of clinical manifestations ranging from localized nummular lesions to generalized exfoliative erythroderma in the most severe cases 132. Adding to the complexity of the clinical picture are the many possible categorizations of AD, such as:

1. infantile/childhood/adolescent/adult stages
2. early onset/late onset
3. transient/persistent
4. acute/subacute/chronic
5. mild/moderate/severe
6. intrinsic (low IgE, 20%)/extrinsic (high IgE, 80%)
7. African/Asian/European American phenotypes
8. ± comorbidities: food allergy/asthma/rhinitis/infections
9. ± genetic risk factors: e.g. FLG mutations
10. ± environmental risk factors: multiple (microbiome/exposome)
11. ± response to a given treatment

Though some of the categories are overlapping (e.g. all infantile stages are early onset), most of them can be combined (e.g. early/late onset × transient/persistent × mild/severe × low/high IgE × ethnicity × comorbidity × ±FLG mutations), resulting in thousands of possible composite classifications. This does not in itself pose a problem, because most of the above features are phenotypic and therefore relatively easy to record. No, what we would like to understand are the underlying disease endotypes. In other words: which molecular features and pathways characterize the different subtypes of AD 201, and can we identify relevant endotype‐specific biomarkers that can predict disease trajectories and guide choice and intensity of treatment? That is the question, and a difficult one indeed, because of the both heterogeneous and complex nature of AD, being the result of multiple genetic, environmental, and immunologic factors. This is reflected in our inadequate understanding of the pathogenesis of AD (outlined in Fig. 2), and the ongoing discussion of whether it is an ‘outside‐in’ (disruption of the epidermal barrier triggers the immune system) or an ‘inside‐out’ (inflammation causes the barrier dysfunction) disease 193, 202. But it is not really an either–or question, because current evidence speaks in favor of *both* the above hypotheses, which are therefore not mutually exclusive. Genetic evidence has established that loss‐of‐function mutations in FLG, the gene encoding filaggrin, an important structural protein in the stratum corneum of the epidermis 203, 204, are the major predisposing factors for AD 138. This has been confirmed by twin studies 140, and GWAS data 141, showing that FLG mutations, which are present in about 10% of the population, could stratify for the early‐onset persistent subphenotype in children 154. Between 20 and 50% of moderate‐to‐severe AD patients carry FLG mutations 136, so this AD subset fits well with the outside‐in hypothesis for initiation of AD. But what then, about the other half of AD patients who do not harbor any FLG mutations? In these patients, it is plausible that immune dysregulation results in secondary epidermal barrier disruption, in line with the inside‐out hypothesis. Or, alternatively, a combination of other genetic, epigenetic, immunological, and environmental factors – the exposome and microbiome included – may trigger and determine the course of AD, in which case, such compound endotypes may be difficult to tease out.

Still, it is beyond doubt that FLG mutation positive AD patients constitute a ‘true’ endotype, and therefore should be treated accordingly, preferably with the aim of reestablishing and maintaining an intact skin barrier as early as possible. This is necessary to prevent allergic sensitization and with this, development of asthma and allergic rhinitis. Ideally, one would like to perform prenatal diagnostics, i.e. WGS on the fetus’ DNA in order to identify all possible – not only skin disease related – genetic risk factors even before birth. Alternatively, and perhaps more feasibly, WGS of the newborn can provide the same information, albeit a little later. In case mutations in skin barrier genes (like FLG) are detected, early intervention schemes can be applied, such as use of emollients soon after birth 205. In best case, such a personalized preventive strategy may hinder development of AD and its comorbidities (the atopic march) altogether.

**Figure 4:**
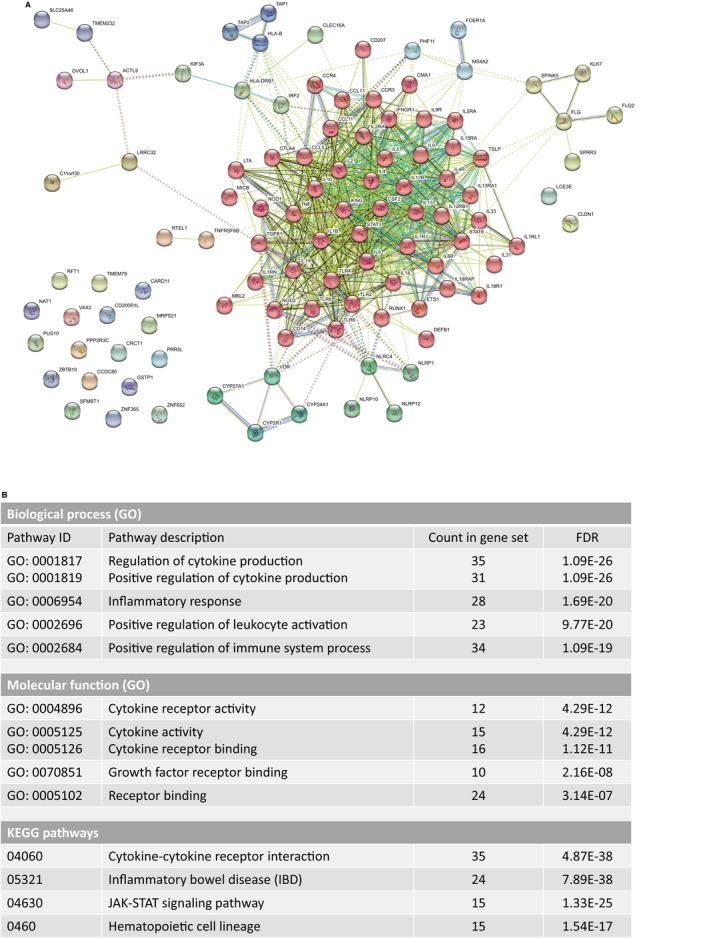
STRING 276 network representation of 105 genes reported to have genetic associations to AD. The genes have been compiled from three publications: Paternoster et al. 2015 (31 loci) 141, Al‐Shobaili et al. 2016 (49 genes) 139, and Liang et al. 2016 (63 genes) 142. The clustering was performed with the ‘MCL inflation parameter’ = 3. The red cluster in the middle of the network represents the cytokine activity enriched gene set. The top enriched biological processes, molecular functions, and KEGG pathways are shown in the table below the network.

Besides FLG mutations, around 100 genes have been identified as AD associated in various studies 139, 141, 142. If one performs a functional enrichment analysis of these genes, it appears that the majority of them are related to inflammation and cytokine activity (Fig. 4), highlighting the potential importance of immune signaling and T‐cell activation in development of AD.

What remains is the detailed analysis of the many possible gene–gene and gene–environment interactions that define the complex endotypes of AD. Here, two genetic variants of particular interest will be mentioned:

CD207: encodes langerin, a pattern recognition receptor expressed in epidermal Langerhans cells (LCs) and involved in antigen‐processing and presentation to T‐cells. Defects in langerin function could therefore have implications for cutaneous immunity, in particular with respect to susceptibility to skin infections by viruses and bacteria like S. aureus 141.

CLDN1: encodes claudin‐1, a tight junction (TJ) protein, expressed by keratinocytes in the stratum granulosum layer of the epidermis and important for maintaining an intact epidermal barrier. In a study from 2011, AD patients (n = 5) were found to have markedly lower expression of CLDN1 compared to healthy controls 206. This finding has been replicated in other studies, showing that CLDN1 expression can be downregulated by IL‐33 via the STAT3 pathway in keratinocytes 207, via IL‐13 in bronchial epithelial tissue 208, and interestingly, that CLDN1 expression can be restored both in human keratinocytes and a murine model of AD by application of the proteasome inhibitor bortezomib 209.

An intriguing link between tight junction function, the recent 2–3‐fold rise in AD prevalence, and the increased use of detergents has been proposed by Dr. Cezmi A. Akdis and colleagues. They demonstrated that even trace concentrations (10^−6^ vol/vol) of commercial detergents were able to directly disrupt tight junctions between keratinocytes in culture and thereby potentially compromise epidermal barrier integrity, thus increasing the risk of allergen penetration and inflammation 210. Although this variant of the hygiene hypothesis is compelling, it remains to be reproduced in an *in vivo* setting on full thickness skin to determine if the detergents can actually penetrate the protective, outermost stratum corneum layer of the epidermis.

### Gene expression analysis – from molecular pathology to targeted therapy

**Figure 5:**
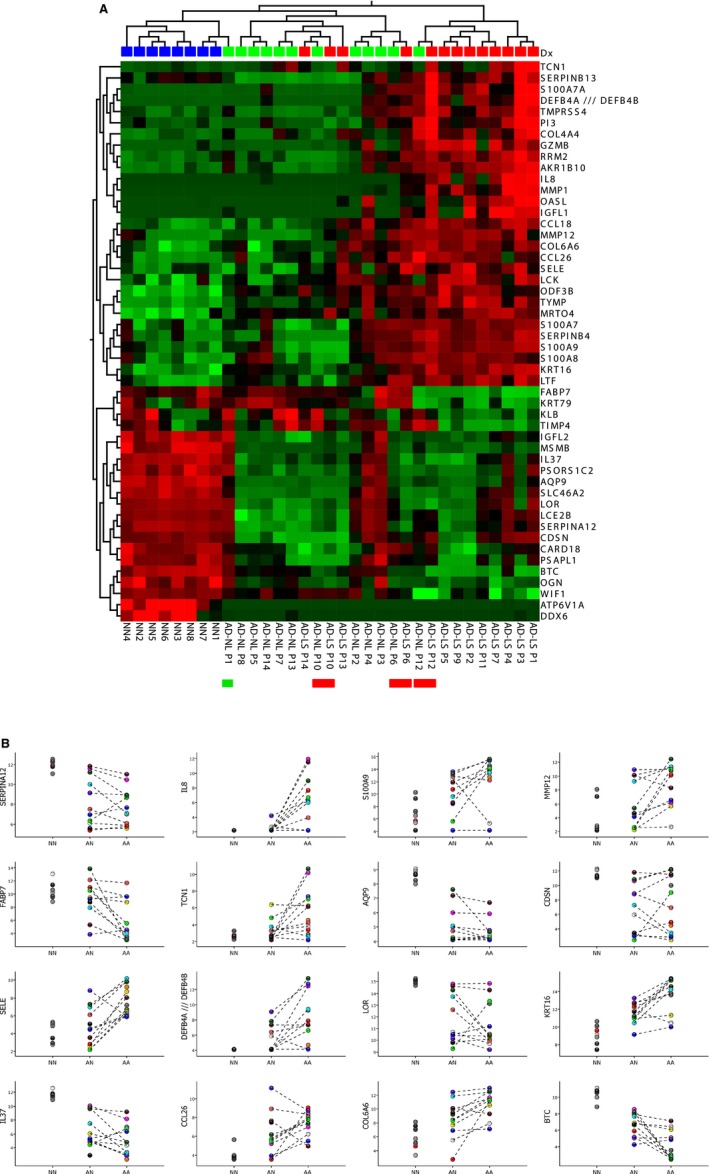
Gene expression analysis of lesional and non‐lesional skin biopsies from 13 AD patients and from eight healthy controls. (A) Heat‐map and two‐way unsupervised hierarchical clustering based on the 50 most variable genes between the three groups (non‐protein coding and orfs (open reading frames) removed). The samples cluster primarily according to disease (AD samples to the right and NN samples in the left cluster) and histology (LS to the right and NL in the middle cluster). What one also can see, is that three of the AD sample pairs (P12, P6, and P10, indicated by red bars below the heat map) cluster together, that is: there are only minor differences between the LS and NL samples from the same patient; the NL P12 sample is ‘lesional’‐like (clusters with the other LS samples), while the three ‘middle‐group’ (having overall low expression of most of the 50 DEG) LS samples (P13, P14, P10) appear more ‘non‐lesional’ like. One NL sample (P1, indicated with a green bar below the heat‐map) clusters with the normal (NN) group, and thus, this AD patient does not appear to have the ‘molecular scar’ typical of non‐lesional AD skin. The colors in the heat‐map signify high (red) or low (green) expression of the particular gene across samples (z‐scaled values). (B) Scatter plots of 16 selected genes, illustrating both the differences between lesional (AA), non‐lesional (AN), and healthy control (NN) samples, and the variability within the groups, revealing the heterogeneity of both the diseased and ‘normal’ (healthy) population. The *Y*‐axis are log2‐transformed expression values (detection limit: 2–4, saturating concentrations: around 15). The samples are colored according to individual, and the dotted lines connect samples (non‐lesional and lesional) originating from the same individual. All the data used for this illustration can be accessed in GEO by its accession number, GSE32924 (https://www.ncbi.nlm.nih.gov/geo/query/acc.cgi?acc=gse32924).

An important information source on the molecular pathology of AD (and any other skin disease) is transcriptomics analysis performed on skin biopsies. Optimally, the biopsies are obtained from site‐matched lesional and non‐lesional AD skin and from healthy – age and gender‐matched – controls. This enables both intra‐individual comparisons (paired analysis of samples from the same subject) and comparisons between the diseased and healthy population. Notably, individual gene expression patterns may expose not only overall disease signatures, but also the heterogeneity (endotypes and sub‐endotypes) of AD, and of the healthy population. This point is illustrated in Fig. 5, which is a re‐analysis of the transcriptomic profile of AD reported by Suárez‐Fariñas et al. 145.

The heat‐map in Fig. 5A is based on the top‐50 DEG, most of which are inflammatory or epidermis associated, and shows that the three histologies (healthy, non‐lesional, lesional) separate, and also, that the separation is not perfect, as several of the lesional (LS) and non‐lesional (NL) samples co‐cluster, which probably reflects the wide disease spectrum of AD. This heterogeneity is further emphasized when one looks at the individual genes (Fig. 5B), which display a striking variability in expression, both between and within groups. For example, IL8 (CXCL8, an inflammatory chemokine, involved in neutrophil activation), MMP12 (expressed by macrophages, degrades elastin), TCN1 (highly expressed in neutrophils), and DEFB4A/B (defensin, expressed by neutrophils and keratinocytes, induced by inflammation) all vary widely in expression, from undetectable to saturating concentrations in AD skin. Also, LOR (loricrin) and CDSN (corneodesmosin), which both are terminal differentiation genes, and believed to be downregulated in AD skin, are seen to have highly variable expression, ranging from undetectable (CDSN) to the high levels also found in normal skin. Also note, that not only AD skin but also healthy skin varies extensively in expression of some of the genes shown, such as MMP12, S100A9, and COL6A6, illustrating the variation within the normal population. The high variability, in particular in AD skin, most likely reflects both the extent of disease – the more inflamed skin, the higher the expression of inflammatory genes – and its pathogenesis, where multiple pathways, some associated with keratinocyte defects, some with immune system dysfunction, may coexist, and where proper (and probably problematic) identification of the underlying, dominant disease endotype eventually may guide targeted therapy.

We and others –‐ in particular the laboratories of Emma Guttman‐Yassky at Mount Sinai and James Krueger at Rockefeller University, both in New York, have generated a number of gene expression studies on skin have deposited transcriptomics data on AD and PSO in the public Gene Expression Omnibus Database GEO 211 for further analysis. Table 1 summarizes a selection of AD and PSO studies of interest and also links to the respective datasets.

A closer look at the above table reveals that several of the aforementioned categorization aspects of AD have already been addressed by transcriptomics analyses, including:

#### Early‐onset AD in children vs adult AD

The skin transcriptome of 19 young children (with no known FLG mutations) with early‐onset AD was compared to that of age‐matched healthy controls, as well as to adult AD patients/controls. In common for both child and adult AD patients were alterations in lipid metabolism and tight junction associated genes as well as Th2‐mediated inflammation 212. In addition, the pediatric patients displayed significant Th17/Th22 polarization, but neither Th1 activation nor downregulation of epidermal differentiation complex genes, which are characteristic features of adult AD. Still, larger cohorts are needed to take ethnic differences (the above study included Asian, African, Hispanic, and Caucasian patients, the former three ethnicities with only 1–2 matching controls) and subgroups with FLG mutations into account. However, because obtaining skin biopsies from children is challenging, in such studies other, less invasive techniques, such as tape‐stripping 213 or blood‐based biomarkers 214, 215, are preferable.

#### Acute vs chronic AD

Here, sequential biopsies were obtained from 10 patients in their acute and chronic phase of AD. Acute lesions were characterized by a marked increase (compared to non‐lesional skin) in expression of epidermal differentiation complex (EDC) genes, in particular S100A7/A8/A9, which are associated with Th2 (IL‐4, IL‐13, IL‐31) and Th22 (IL‐22) cytokine activation 196. When progressing to chronic lesions this Th2/Th22 axis was further activated followed by an increase in Th1‐associated products, such as CXCL9/10/11 (see Fig. 2). In terms of treatment selection, this could point toward targeting Th2/Th22 pathways in acute AD.

The effect of ethnicity on AD has been shown in Asian (Japanese and Korean) patients, who in general display a more psoriasiform AD phenotype and significantly higher Th17/Th22 activation as assessed by cytokine expression (IL‐17A, IL‐19, IL‐22, S100A12), compared to European American AD patients 212, 216. This can have implications for the choice of treatment, as the selective blockade of IL‐17/IL‐22 pathways could be indicated in the Asian, ‘psoriasis‐like’ immune phenotype. It will be interesting to see if the reported Japanese/Korean AD phenotype extends to the larger Chinese and Indian populations, and also, to include migration studies (investigating Asian American, Asian European, as well as local Asian AD) to evaluate the genetic, epigenetic, and environmental (exposome/microbiome) effects on the development of AD. The above considerations of course also apply to other non‐European ethnic groups, including the African population, who is likely to have yet other genetic susceptibilities, as recently reviewed by Kaufman et al. 217 and Brunner et al. 218.

A transcriptomic treatment response signature has been obtained in several clinical intervention studies, such as ultraviolet B (UVB) phototherapy 157, cyclosporin A 158, dupilumab 159, and fezakinumab 161. In these studies, pre‐ and post‐treatment skin biopsies were obtained from both lesional (AD‐LS) and non‐lesional (AD‐NL) skin. The number of differentially expressed genes (DEGs) between AD‐LS and AD‐NL was found to be lower post‐treatment compared to pre‐treatment, indicating a normalization of the AD disease signature, including suppression of Th1, Th2, and Th22 inflammatory markers 157. However, because not all genes improve, even after successful clinical remission as assessed by SCORAD, they are defined as comprising a *residual disease genomic profile* (RDGP) 219. The RDGP concept was originally introduced when the treatment of psoriasis with etanercept resulted in the resolution of disease and normalization of many, but not all psoriasis‐related genes 220. A subset of 248 genes did not return to baseline (or rather: exhibited less than 75% improvement after treatment) and could be indicative both of incomplete suppression of inflammation – leaving room for improvement – and for a ‘molecular scar’ intrinsic to the disease. Whether the latter represents different endotypes with implications for disease progression and treatment response remains to be determined.

A meta‐analysis derived AD (MADAD) signature identified 595 AD‐associated DEGs across four publicly available transcriptomics studies 146, and a subset of the most discriminatory of these genes was shown to be applicable as a robust standardized measure of treatment effect in the abovementioned UVB, cyclosporin A, and dupilumab studies. Since the MADAD reference transcriptome captures both immunological (inflammatory genes, cytokines, T‐cell receptor signaling) and barrier defect (epidermal differentiation, lipid metabolism) genes, it may be used for future evaluation of therapeutic response.

**Figure 6:**
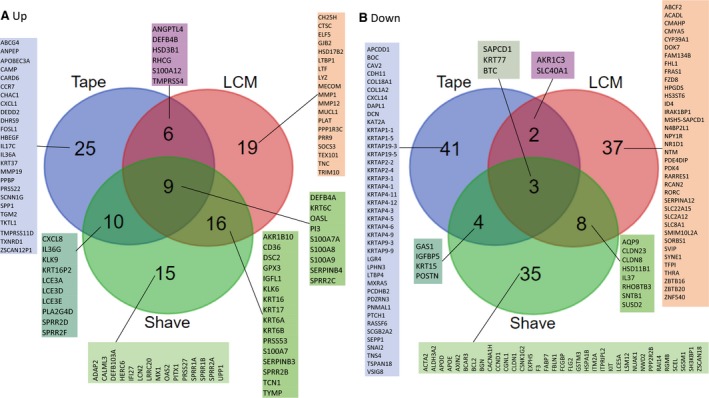
Comparison of three studies, assessing the epidermal transcriptomic profile of AD‐LS vs AD‐NL skin. (A) Top‐50 up DEG (LS vs NL). (B) Top‐50 down DEG (LS vs NL). The three studies included are Tape: Tape‐stripped skin (not in GEO) 213; LCM: GSE120721 222; Shave: GSE60709 223. For the genes that are up in LS/NL epidermis, the overlap in DEG between LCM and epidermal shave is 50% (25 out of 50 genes). Fewer genes are in common between the three studies for the down DEG. For the tape‐stripping study, interestingly, many KRTAP genes appear as lower expressed in LS skin. Since KRTAP genes are associated with the hair shaft, this could suggest that there is less hair in the LS epidermis region.

All of the above transcriptomic studies have been performed on full thickness punch biopsies of the skin, which is organized in three main layers: the epidermis, the dermis, and the subcutaneous fat (hypodermis). The epidermis contains >90% keratinocytes at different differentiation levels, a few melanocytes, Langerhans cells (LCs), Merkel cells, α‐dendritic cells, and inflammatory cells. The dermis mainly consists of extracellular matrix proteins, primarily collagen fibers produced by fibroblasts, and also dendritic cells/macrophages, mast cells, various unactivated/activated T‐cell subsets, plasma cells, hair follicles, sweat glands, sebaceous and apocrine glands, and endothelial cells 221. Thus, when we analyze gene expression in whole skin, the resulting average signal will reflect both the cellular distribution, such as the epidermis‐to‐dermis ratio, which is known to vary both in AD and PSO, as well as any up‐ or down‐regulation of differentially expressed genes. Furthermore, in a homogeneous assay the compartmental localization of gene expression is lost and also the expression of low‐abundance genes may become undetectable because of dilution. One way to generate a more refined skin transcriptome is to apply LCM (laser capture microdissection), which enables separation of the skin into its dermal and epidermal components. Such a – and so far only – study based on paired lesional and non‐lesional samples from five AD patients showed that indeed the dermal and epidermal transcriptomes differ, and also, that the AD signature could be expanded with some 1000 DEGs due to the increased signal‐to‐noise ratio, when working with separate compartments 222. Some of the DEGs now detectable by LCM included IL22, TSLP, IL34, CCL22, CCL26, CLDN4, and CLDN8 (the latter two involved in tight junction (TJ) formation). One reason why LCM is not applied routinely is that it is very labor intensive. Therefore, other methods need to be considered for separating the dermal and epidermal signals. One such method is based on epidermal shaves for transcriptomic profiling 223, another is tape stripping 213. A quick comparison of the top‐50 LS/NL DEGs from each of the three studies shows that they have only little overlap (Fig. 6). This may be partly due to different patient populations, different skin sampling technologies, and different RNA quantification platforms (Affymetrix microarrays, Illumina arrays, Ion Torrent sequencing). In particular, the tape‐stripping experiment displayed some unexpected findings, such as large differences in the expression of numerous keratin‐associated protein (KRTAP) genes (being more than 50‐fold downregulated in LS vs NL epidermis), which could suggest differences in the presence of hair.

### Microbiome

Both the gut and skin microbiome have been implicated in the pathogenesis of AD.

The gut microbiome is associated with the *hygiene hypothesis* proposed by Strachan in 1989, who noticed that the number of older children in a household had a striking inverse correlation to the prevalence of hay fever and eczema in their younger siblings 128. Thus, he hypothesized that smaller family size leads to less unhygienic contact with older siblings and thus, reduced the opportunity for cross infection that supposedly has a protective effect on development of eczema. In addition, higher microbial endotoxin exposure from farm animals was associated with protection against development of allergy, as suggested in the ‘Eat Dirt’ article by Weiss 224. Finally, because the gut microbiome is important for the prenatal–early‐life maturation of our immune system 225, it has been intensively studied in relation to allergic diseases, and perturbations in the infant gut microbiome have been linked to the risk of developing AD 226. However, several probiotic and prebiotic intervention trials have been conducted, and although some protective effect against AD was observed, the differences were small, and no clear gut microbiome–AD association could be demonstrated 227.

The cutaneous microbiome, on the other hand, is clearly associated with AD, as a vast majority (>90%) of AD patients have their skin colonized with Staphylococcus aureus, compared to only 5% of healthy controls 132. In addition, S. aureus positive AD patients seem to have more severe disease, higher levels of Type 2 biomarkers (CCL17, POSTN), allergen sensitization (IgE), and barrier dysfunction (higher TEWL) than non‐colonized AD controls 228. Also, in a prospective study on children with AD, an increase in the proportion of S. aureus and a concomitant decrease in bacterial diversity preceded worsening (flare) of AD 229. To address the question as to whether the observed colonization of AD skin by S. aureus is potentially driving the disease or merely an epiphenomenon, several intervention trials have been carried out. Such studies applying either antiseptics or antibiotics have demonstrated that a reduction in the level of S. aureus is indeed followed by a reduction in AD severity, further supporting a causal relationship between S. aureus colonization of skin and AD 227. Additionally, use of emollients in infants at risk for developing AD caused a decrease in skin pH and an increase in bacterial diversity, which may partly explain the preventative effects of emollients 230.

A mechanistic link to the pathogenesis of AD is suggested by the colonization of AD skin by toxigenic S. aureus strains that produce *superantigens* (SA), which drive the development of a Th2 immune response, and activate Langerhans cells (LCs) and cutaneous dendritic cells (DCs) that bridge innate and adaptive immunity 231. Moreover, mast cell degranulation is directly stimulated by the S. aureus δ‐toxin (δ‐hemolysin), which also promotes IgE production and Th2‐mediated inflammation 232. Conversely, Th2 cells enable S. aureus colonization due to IL‐4 and IL‐13 mediated inhibition of antimicrobial peptides (AMPs) and of terminal differentiation proteins important for skin barrier integrity 233. And in turn, S. aureus amplifies the inflammatory response by stimulating release of IL‐4, IL‐13, IL‐22, IL‐17, IL‐31, and IgE, thus closing the vicious circle 234. It will be interesting to see whether the topical application of protective commensal skin bacteria, such as coagulase negative Staphylococcus strains, can inhibit the growth of S. aureus and eventually, lead to a cure of AD 235 (Box 5).

Box 5 **Technical considerations – what to sample and what to measure?**

As the eyes are said to be the mirror of our soul, so is the skin a two‐way mirror,^18^ reflecting both our inner and outer environment 237. Therefore, the cutaneous inflammation observed in AD reflects both inherent skin barrier and immune system dysregulation (endotype) as well as the effect of external factors (exposome), such as allergens, bacterial toxins, detergents, and other irritants.

Fortunately, the skin is easily accessible, and as such, straightforward to sample and study by various techniques, from imaging (phenotype) to ‐omics analyses (molecular pathology) on biopsies. Standard 3–4 mm biopsies, however, damage the skin and are considered too invasive for routine use on children, who represent the majority of AD patients, which is why alternative sampling technologies should be considered. One alternative, is to take smaller, 1 mm ‘mini’ biopsies (for which commercial punches are available) with only minimal scarring, allowing for multiple biopsies to be sampled, but which still require the application of local anesthetics, as otherwise, the procedure is painful. Another, less invasive option is to apply tape stripping to remove the stratum corneum, enabling quick sampling of multiple epidermal layers. A third, and non‐invasive technology is the procedure of skin surface washings that within 30 min sampling time allows for quantification of stratum corneum associated cytokines 237.

Blood sampling is of course obvious, but first requires identification of adequate biomarkers 215. The advantage of a blood sample is that it can integrate the disease signature, and that it contains several subsets of inflammatory cells that can be identified, separated, and analyzed by flow cytometry. A disadvantage of blood‐based biomarkers is the dilution effect; if local skin inflammation is the dominating disease feature, it may be difficult to translate into a blood‐based biomarker.

Other sampling sites include urine 238 and exhaled breath condensate, the latter containing markers of airway inflammation, which has been reported in children with AD 239.

A note on the analysis of skin biopsies: when we take a full thickness skin biopsy, it contains a heterogeneous mixture of cells, which are being homogenized before DNA, RNA, or protein is extracted for further analysis. Thus, the sample is blended into a ‘cellular smoothie’, where the individual cell characteristics are evened out or even lost. Thus, what we see, when we analyze the gene expression pattern from such a homogenized biopsy, is a snapshot in time and space; it is an average signal from many (millions) individual cells and several cell types, but where the individual, cell‐specific signals are averaged out. The problem is that we do not know the distribution of different cell types in the sample. Thus, we cannot tell whether the actual – average – signal measured is due to activation or inhibition of specific genes (up‐ or down‐regulation of gene expression), or whether it is due to redistribution of compartments. For example, FLG and LOR are highly expressed in the epidermis and are often seen to decrease following perturbation. But whether the observed differential gene expression is due to inhibition, or whether it is because of thinning of the epidermis (thus, decreasing the epidermis to dermis ratio) remains unknown. One way of solving the problem is by deconvolution of the cellular compartments, i.e. estimating the percentage of epidermis, dermis, and other skin compartments based on tissue‐ and cell type‐specific expression patterns 240, 241, 242. This ‘housekeeping’ approach may work in a well‐defined system, but such a system is rarely well defined. The cell‐specific signals can be identified by laser capture microdissection (LCM), which enables separation of the dermis from the epidermis signal, and thus increases the signal‐to‐noise ratio compared to that of a full thickness biopsy 222. LCM is rarely applied, however, mainly because it is a very laborsome technique, and also because it can affect the actual state of the cells 243. Another, more high‐throughput technique, is separation by e.g. microfluidics or flow cytometry into single cells followed by single‐cell sequencing, for example, by drop‐seq, made open source by Steve McCarroll et al. (http://mccarrolllab.org/dropseq/). Thus, instead of a cellular smoothie, we now have a cellular fruit salad, where the characteristics of each individual piece of fruit are retained 244.^19^

### How precise is precision medicine?

Usually, differences between diseased and healthy tissue are quantitative rather than qualitative. That is: a given target or pathway is rarely exclusive to just a single disease, tissue, or cell type. For example, cytokines and their receptors are expressed at highly variable levels across cell types and conditions, and while certain cells, such as Th2 cells, express and release high levels of IL‐4 and IL‐13, this is also the case for basophils and ILC2 cells 245. And conversely: the expression of cytokine receptors, e.g. the IL‐4Ra subunit, is not confined to just a single‐cell type, like keratinocytes (which themselves secrete numerous cytokines that act as both autocrine and paracrine mediators). Therefore, targeting the IL‐4 receptor pathway with a specific antibody like dupilumab may cause adverse effects – such as the conjunctivitis reported in 14–19% of AD patients treated with dupilumab 246. Of course, this concern also applies to any other targeted treatment, which is why it is important to evaluate and prioritize both the most relevant disease drivers, pathways, and targets (druggability considerations) as well as to take potential off‐target and on‐target adverse effects into consideration.

Finally, to evaluate the efficacy of novel medicine approaches and to identify the most important disease pathways, head‐to‐head comparisons against other targeted treatments are useful. A notable example of such a head‐to‐head comparison is the recent ECLIPSE study including 1048 moderate–severe PSO patients. In this Phase 3 study, the long‐term efficacy and safety of the IL‐17A inhibitor secukinumab was compared to that of the IL‐23 (p19) inhibitor guselkumab: at week 48, PASI90 was reached for 70% of patients on secukinumab, and for 85% of patients on guselkumab, while the PASI100 responses were 48% and 58%, respectively 247. Thus, for this patient group, the newer‐generation IL‐23 inhibitor demonstrated superior long‐term efficacy over the IL‐17A inhibitor.

In another head‐to‐head PSO study, the IL‐23 (p19) inhibitor risankizumab was compared to the dual IL‐12/23 inhibitor ustekinumab, and already at week 4 the p19 inhibitor showed a more pronounced effect than ustekinumab as assessed by molecular (RNA‐seq transcriptomics) and histopathologic profiling 153.

In a third PSO study, the effect of an anti‐IFN‐γ antibody was investigated, and although IFN‐γ was blocked at the molecular level, clinical efficacy could not be demonstrated 248. This – obviously – implies that it is not sufficient to demonstrate the molecular effect of target inhibition, if the target is not central for driving the disease.

### Personalized medicine – strategies and candidates

Targeting signaling pathways in inflammatory skin diseases can be approached at different cellular levels: *extracellularly*, at the receptor level, either targeting the cytokine itself (e.g. tralokinumab for IL‐13), or its receptor (e.g. dupilumab for IL‐4RA) by monoclonal antibodies (or other biologics/biosimilars, such as nanobodies) or, alternatively, *intracellularly*, blocking the downstream signaling of e.g. the JAK‐STAT pathway (Fig. 3B‐C) by small‐molecule inhibitors of one or more of the four JAKs: JAK1, JAK2, JAK3, and TYK2 249.

Currently, several JAK inhibitors – both systemic and topical –with different selectivities are in clinical development for treatment of inflammatory diseases (see Table 2 for these and other compounds), including filgotinib (JAK1), upadacitinib (JAK1), abrocitinib (JAK1), ruxolitinib & baricitinb (JAK1/2), tofacitinib (JAK1/3), BMS‐986165 (TYK2), ASN002 (TYK2/SYK), and delgocitinib (JTE‐052, pan‐JAK). Most of these candidates show promising efficacy and overlapping systemic safety profiles with increased risk of opportunistic virus infections and cytopenias 250. Therefore, to avoid the latter adverse effects of systemic treatment, topical formulations should also be (and are) considered.

**Table 2:** Future and emerging targeted treatment options for atopic dermatitis (AD) and psoriasis (PSO)

Target	Drug	Literature reference	clinicaltrials.gov reference^a^	Indications	Clinical phase, status
AhR (agonist)	Tapinarof (t)^b^ (GSK2894512)	294, 295	NCT02564055 NCT03202004	AD PSO	Ph.II, completed Ph.III, withdrawn
CCL20	GSK3050002 (i)	296	NCT02671188	PsA	Ph.I, withdrawn
CD125 (IL5RA)	Benralizumab (sc)	297	NCT03563066	AD	Ph.II, recruiting
H4R	ZPL‐389 (o)	294	NCT03517566 NCT02618616	AD PSO	Ph.II, recruiting Ph.II, completed
IgE	Omalizumab (sc) QGE031 (sc)	298	NCT02300701 NCT01552629	AD pediatric AD	Ph.IV, active, Ph.II, completed
IL‐1a	Bermekimab (sc)		NCT03496974	AD	AD: Ph.2, recruiting
IL‐4Ra	Dupilumab (sc) (Dupixent)	200, 299	NCT02612454 FDA approved	AD pediatric	Ph.III, enrolling
IL‐5	Mepolizumab (sc)	300	NCT03055195	AD	Ph.I, terminated
IL‐12B (p40)	Ustekinumab (Stelara) (sc)	301 302, 303	NCT01806662 NCT02698475	AD PSO	Ph.II, completed Ph.III, pediatric PSO
IL‐13	Tralokinumab (sc)	304	NCT03587805	AD	Ph.III, recruiting
IL‐13	Lebrikizumab (sc)	299, 305	NCT03443024	AD	Ph.II, active
IL‐17A	Ixekizumab (sc)	306	NCT03073200	PSO	Ph.III, pediatric PSO
IL‐17A	Secukinumab (sc)	262 303	NCT02594098 NCT02409667	AD PSO	Ph.II, completed Ph.III, completed
IL‐17A/IL‐17F	Bimekizumab (sc)	307	NCT03598790	PSO	Ph.III, recruiting
IL‐17C	MOR106 (iv)	308	NCT03568071	AD	Ph.II, recruiting
IL‐17RA	Brodalumab (sc)	174	NCT03403036	PSO	Ph.IV, completed
IL‐22	Fezakinumab (sc)	161	NCT01941537	AD	Ph.II, active
IL‐23 (p19)	Guselkumab (sc) Tildrakizumab (sc)	309 169	NCT02905331 NCT01729754	PSO	Ph.III, completed Ph.III, active
IL‐23 (p19)	Risankizumab (sc) Mirikizumab (sc)	310 311	NCT03518047 NCT03482011	PSO	Ph.III, active Ph.III, recruiting
IL‐31	BMS‐981164 (sc)	312	NCT01614756	AD	Ph.I, completed
IL‐31RA	Nemolizumab (sc)	275	NCT03100344	AD	Ph.II, completed
IL‐33	Etokimab (sc) (ANB020)		NCT03533751	AD	Ph.II, recruiting
IL‐36R	ANB019 (sc)		NCT03619902	PSO (GPP)	Ph.II, recruiting
JAK1	Upadacitinib (o) Abrocitinib (o) (PF‐04965842)	313 299	NCT03607422 NCT03575871	AD	Ph.III, recruiting Ph.III, recruiting
JAK1/2	Ruxolitinib (t)	314 315 316	NCT03745638 NCT00820950 NCT02553330 NCT03099304	AD PSO AA Vitiligo	Ph.III, recruiting Ph.II, completed Ph.II, terminated Ph.II, recruiting
JAK1/2	Baricitinib (o)	317, 318	NCT03334422 NCT01490632	AD PSO	Ph.III, active Ph.II, completed
JAK1/3	Tofacitinib (o,t)	314 319 315	NCT02001181 NCT01241591 NCT02812342	AD PSO AA	Ph.II, completed Ph.III, completed Ph.II, active
JAK1/TYK2	PF‐06700841 (o)	320	NCT02969018	PSO	Ph.II, completed
JAK1/2/3, TYK2	Delgocitinib (t) (JTE‐052)	321	NCT03725722	AD	Ph.II, recruiting
NK‐1R (TACR1)	Serlopitant (o)	322	NCT02975206	AD, pruritus	Ph.II, completed
NK‐1R (Substance P)	Tradipitant (o)	322	NCT03568331	AD, pruritus	Ph.III, recruiting
OX40	GBR 830 (sc)	323	NCT03568162	AD	Ph.II, recruiting
OX40	KHK4083 (i)		NCT03096223	AD	Ph.I, completed
PDE4	Apremilast (o)	324 325	NCT02087943 NCT01194219	AD PSO	Ph.II, completed Ph.III, completed
PDE4	Crisaborole (t)	326 327	NCT02118766 NCT01300052	AD PSO	Ph.III, completed Ph.II, completed
PDE4	OPA‐15406 (t)	328	NCT02068352	AD	Ph.II, completed
RIP1 kinase	GSK2982772 (o)	329	NCT02776033	PSO	Ph.II, completed
ROR‐γ	ESR‐114 (t)		NCT03630939	PSO	Ph.II, recruiting
SYK/JAK	ASN002 (o)		NCT03531957	AD	Ph.II, recruiting
TNF‐α	Infliximab (i)	169	NCT00686595	PSO, PsA	Ph.IV, completed
TNF‐α	Adalimumab (Humira) (sc) Etanercept (sc)	330	NCT01970488 NCT00332332	PSO	Ph.III, completed Ph.IV, completed
TNF‐α/IL‐17A	ABT‐122 (sc)	331	NCT02349451	PsA	Ph.II, completed
TNF‐α/IL‐17A	COVA322 (i)	183, 332	NCT02243787	PSO	Ph.I, terminated (safety)
TrkA	CT327/SNA‐120 (t)	322	NCT01808157	AD, pruritus	Ph.II, completed
TRPV1	PAC‐14028 (t)	333	NCT02748993	AD, pruritus	Ph.II, completed
TSLP	Tezepelumab (sc) (AMG 157)	323	NCT00757042	AD	Ph.I, completed
TYK2	BMS‐986165 (o)	178	NCT03624127	PSO, PsA	Ph.III, recruiting

Sorted according to molecular target. Monoclonal antibody drugs can be identified by their names, which all end with ‘‐mab’.

a The list is not exhaustive. For up‐to‐date information on the clinical trials, please see https://clinicaltrials.gov

b (t) topical; (iv) intravenously; (o) oral; (sc) subcutaneously.

Finally, new treatment modalities include those based on vaccination and allergen‐specific immunotherapy. The latter is effective in treating allergies and involves de‐sensitization via repeated exposure to increasing doses of allergens, but the effect on AD is still unresolved 251. Vaccination against S. aureus could in principle eliminate this pathogenic factor from susceptible AD patients, and several clinical trials are ongoing to evaluate the effect of active and passive vaccine candidates on AD 252.

## Conclusion

Historically, we have moved from ignorance, superstition (or act of God) and metaphysics, to a rational (Hippocratic), physical approach to personalized medicine, driven by major progress in technology; this has enabled us to zoom in, both on the cellular and the molecular (the omics revolution) basis of disease. And now – in the post‐genomic era, we are able to integrate the multiple levels of information: from molecular‐level genome, epigenome, metabolome, and proteome data, to higher level physiome, exposome, microbiome, and interactome data 253. Thus, we are aiming at a modern, systemic (systems biology), holistic disease understanding, where the gap between diagnostics and treatment options is steadily closing.

Oncology is leading the way in precision medicine 254, though for a critical review of precision oncology, see Brock and Huang 255. Immunology is catching up with asthma ahead, already linking phenotypes and endotypes to targeted therapy 256, and as a natural extension of this development, inflammatory skin diseases follow suit, with PSO ahead of AD 197.

**Figure 7:**
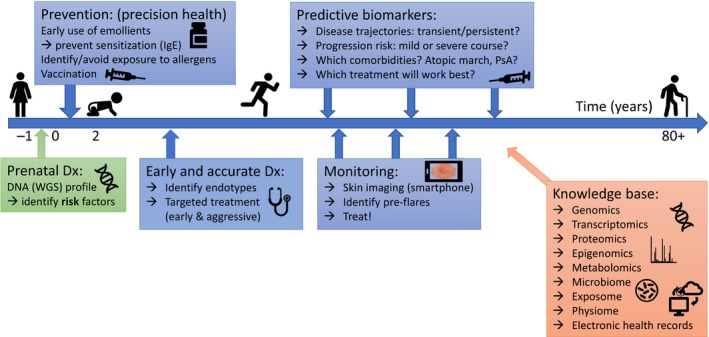
The vision of applied personalized medicine in inflammatory skin diseases. Risk factors (such as FLG mutations in AD) can be identified before birth, enabling preventive measures (such as use of emollients) in early childhood. Identification of endotypes can guide targeted treatment, and a combination of predictive biomarkers and skin monitoring (aided by machine learning, including AI, integrating the information knowledge base) may help identify pre‐flares and optimal time and type of treatment.

In principle, chronic inflammatory skin diseases, like PSO and AD, and also alopecia and vitiligo, are (currently) incurable, but they do respond to treatment. They also, in particular AD, comprise complex and heterogeneous underlying endotypes, which are good candidates for a personalized medicine strategy. Thus, in order to apply endotype‐driven strategies for stratification and personalized medicine, it is necessary first to identify and understand these endotypes. Hopefully, such understanding can be obtained via an integrative, multi‐omics approach resulting in discovery of molecular biomarkers, both prognostic and predictive, for assessing the likelihood of comorbidity, disease progression, and response to novel, targeted treatments (Fig. 7). Additionally, and following the vision of P4 medicine being both personalized and participatory, patients will have the opportunity to monitor the health of their skin by using mobile apps 257 that in real‐time (by use of artificial intelligence (AI) and cloud‐based deep learning) can perform image analysis, evaluate the degree of treatment response, and eventually, recommend to stop, continue, or change the treatment, essentially enabling truly individualized medicine (Box 6).

Box 6 **Key messages**

As our mechanistic understanding of inflammatory skin conditions like PSO and AD increases, so does the potential for personalized treatment and prevention. In particular, because PSO and AD are both complex and heterogeneous diseases with variable course, treatment response, and hard to predict comorbidities, they pose paradigmatic obvious cases for a personalized medicine approach.

Inflammatory skin diseases are currently incurable, but not intractable.

Ideally, personalized management of PSO and AD is *patient‐centric*, i.e. taking the individual's needs into concern. Targeted treatment of the skin with emollients and topical corticosteroids may be sufficient to control disease in mild to moderate cases. Targeted, antibody‐based therapies have revolutionized the treatment of severe PSO and AD, and still more efficient (more patients reaching PASI 100/EASI 100) and safe medicines are in development.

Targeted therapies need to be tailored to the endotypes of AD, and thus, depend on identification of relevant biomarkers of the underlying pathways that drive the disease.

The move from personalized medicine to precision health can be achieved by early intervention (‘treat early and hard’) and prevention (see Fig. 7). Strategies include vaccination and avoidance of triggering factors in predisposed individuals, who can be identified even before birth by genotyping their DNA.

The author would like to acknowledge Hanne Norsgaard, Paola Lovato, Jakob Felding, and Witte Rush Koopman Jr. for insightful comments and suggestions for improvements, Adrian Ewald and Mette Vesterager for rhetorical sparring, and Michala Litman for lending a scratching hand (Fig. 2).

## Notes

1 And for less than 400 EUR. At this price for WGS, direct‐to‐consumer genomics is a reality.

2 and is extremely useful for stratifying according to pharmacokinetics (responders/non‐responders, fast/slow metabolizers, etc.), risk factors and targets [such as the around 300 driver genes and 3400+ driver mutations identified in cancer, crucial for precision oncology 30], and an absolute prerequisite for pharmacogenomics.

3 Endotype: a subtype of a disease or a subgroup of a population defined by a shared, underlying disease mechanism 31. Diseases like asthma 32 and AD 33 encompass several endotypes (for example, those with or without filaggrin mutations), each with their characteristic, underlying pathophysiology. This is in contrast to the Phenotype: the appearance of a disease or an individual, i.e. its observable features or traits (size, shape, pattern, color, behavior, etc.). The phenotype is determined by the sum of interactions between genotype, epigenetic factors, and the environment. Endophenotype: Also known as an ‘intermediate phenotype’ is a quantitative, biologic characteristic that lies between the phenotype and genotype, but is mechanistically closer to the disease than its clinical phenotype; in this context, it corresponds to a biomarker profile 34.

4 Again, Hippocrates should be recognized for introducing the central concept of prognostics, about which he remarked: ‘he will manage the cure best who foresees what is to happen from the present state of matters. For it is impossible to make all the sick well.’ 39

5 Sometimes such identification of responders (theratypes) to therapy is also called theratyping 41.

6 or ‘panoromic’, a term coined by Eric Topol to emphasize the wide‐angle view of multiple approaches 22, although one could argue that it is not only a wide, but also a *deep* view across layers.

7 less than 200 EUR, including strand‐specific library preparation and 30 million paired‐end reads per sample

8 The same consideration applies to ‘standard’ gene expression analysis, where the observed differential gene expression between two samples can be due to both redistribution of different compartments and to actual up‐ or down‐regulation of gene expression.

9 This EWAS acronym is not to be confused with the epigenome‐wide association studies mentioned before.

10 The principles for personalized medicine outlined for PSO and AD also apply to other inflammatory skin diseases, including allergic conditions such as contact dermatitis and urticaria, and autoimmune diseases like vitiligo, alopecia areata, and lichen planus.

11 Based on a world population estimate of 7.7 billion 124 of which 26% are children of age 0–14.

12 Because genetic changes take much longer (evolutionary) time to manifest. In addition, increased disease awareness (such as access to the Internet) may also partly explain the increase in prevalence.

13 According to opentargets.org

14 PASI 75 is a response rate and indicates the percentage of patients who have reached a 75% or more improvement (reduction) in their Psoriasis Area and Severity Index (PASI) score compared to baseline. PASI is a quantitative, composite measure of the severity and extent of psoriatic lesions taking into account erythema, thickness, and scaling of the lesions as well as percentage area affected. It is the most widely used tool in clinical trials for assessing psoriasis severity, although alternative, simpler to apply approaches, such as the Physician Global Assessment (PGA), have recently been proposed 170. As treatments become more effective, higher response rates are often reported, including PASI 90 and even PASI 100 (complete clearance of all disease) 171.

15 Note, however, that accumulation of neutrophils is not limited to pustular forms of psoriasis, as *Munro's microabscesses* containing large collections of neutrophils in the stratum corneum of psoriasis vulgaris are considered a hallmark of PSO.

16 Albeit promising, the dual IL‐17A/TNF inhibitors have been terminated in development due to safety concerns.

17 EASI 75 is the percentage of patients who have reached a 75% or more reduction in their Eczema Area and Severity Index (EASI) score compared to baseline, in this case after 16 weeks of treatment. Other commonly used clinical measures of AD severity include SCORAD (Severity Scoring of Atopic Dermatitis, which includes both objective physician estimates of disease severity as well as subjective patient estimates of itch and sleep loss), and IGA (Investigator's Global Assessment), and because the three scores complement each other, it has been recommended to apply at least two independent assessment schemes for a reliable evaluation of AD 198. What is not captured by the above scores is the actual disease burden on QoL (quality of life) of AD patients, which is why several questionnaire‐based schemes have been proposed for a standardized QoL assessment, including Patient‐Oriented Eczema Measure (POEM), Dermatology Life Quality Index (DLQI), ItchyQOL, and 5‐dimensions (5‐D) itch scales, all of which show good validity and internal consistency 199. Another important aspect when assessing the clinical efficacy of treatment is the placebo effect, which is notoriously high in AD, and which should therefore always be compared to the drug effect. For example, EASI 50 for the placebo arm was 35% compared to 85% for dupilumab in an early‐phase 12‐week study 200.

18 And a very large mirror indeed; while the conservative estimate of skin surface area is 2 m^2^, then, if one takes the presence of hair follicles, sweat, and sebaceous glands into account, the total skin area – and thus the interface for interaction with the skin microbiome – is probably closer to 30 m^2^, as pointed out by Richard Gallo 236.

19 Please see this inspiring TED talk by Steve McCarroll for the fruit analogy: https://www.ted.com/talks/steve_mccarroll_how_data_is_helping_us_unravel_the_mysteries_of_the_brain
